# Rostral and body shape analyses reveal cryptic diversity of Late Jurassic batomorphs (Chondrichthyes, Elasmobranchii) from Europe

**DOI:** 10.1002/spp2.1552

**Published:** 2024-03-19

**Authors:** Julia Türtscher, Patrick L. Jambura, Eduardo Villalobos-Segura, Faviel A. López-Romero, Charlie J. Underwood, Detlev Thies, Bruce Lauer, René Lauer, Jürgen Kriwet

**Affiliations:** 1 Department of Palaeontology, Faculty of Earth Sciences, Geography & Astronomy, University of Vienna, Josef-Holaubek-Platz 2, 1090 Vienna, Austria; 2 Vienna Doctoral School of Ecology & Evolution (VDSEE), University of Vienna, Djerassiplatz 1, 1030 Vienna, Austria; 3 School of Earth Sciences, Birkbeck College, Malet Street, London WC1E 7HX, UK; 4 Zentrale Einrichtung für Weiterbildung, Leibniz Universität Hannover, Schloßwender Straße 5+7, 30159 Hannover, Germany; 5 Lauer Foundation for Paleontology, Science & Education, Wheaton, ILs, USA

**Keywords:** geometric morphometrics, Batomorphii, Spathobatidae, body shape, cryptic species, *Aellopobatis bavarica*.

## Abstract

The fossil record of chondrichthyans (chimaeras, sharks, rays and skates) consists largely of isolated teeth, with holomorphic specimens being extraordinary exceptions. However, numerous of these more or less completely preserved specimens are known from several Upper Jurassic deposits of Europe, enabling detailed analysis of their morphology. Batomorphs (rays and skates) resembling modern guitarfishes and wedgefishes (Rhinopristiformes) are among the most common Jurassic chondrichthyans found, but they have been only sporadically studied up to now, resulting in large knowledge gaps concerning their taxonomy and phylogeny. Here, we present the most detailed revision of Late Jurassic holomorphic batomorphs to date, quantitatively analysing body proportions of specimens from Germany (Solnhofen Archipelago), France (Cerin) and the UK (Kimmeridge), using both geometric and traditional morphometrics. Furthermore, we identify qualitative morphological characters for species discrimination, to clarify the taxonomic identity and diversity of Late Jurassic batomorphs based on holomorphic specimens. Our results support the validity of *Belemnobatis sismondae, Kimmerobatis etchesi* and *Spathobatis bugesiacus*, as well as that of the previously doubtful *Asterodermus platypterus*. Moreover, we describe *Aellopobatis bavarica*, a new taxon, which has hitherto been considered to be a large-sized morphotype of *Spathobatis bugesiacus*. Our results highlight that the diversity of holomorphic batomorphs during the Late Jurassic was greater than previously thought, and suggest that this group was already well-established and diverse by this time. This study thus provides vital information about the evolutionary history of Late Jurassic batomorphs and has direct implications for batomorph species that are based on isolated teeth only.

Chondrichthyans or cartilaginous fishes, the sister group of all other jawed vertebrates, include chimaeras, sharks and batomorphs (rays and skates), with batomorphs being the largest living group, comprising more than half of all extant chondrichthyan species (Fricke *et al*. 2022). Currently, extant batomorphs are arranged in four orders, Myliobatiformes (stingrays and allies), Rhinopristiformes (shovelnose rays and allies), Torpediniformes (electric rays) and Rajiformes (skates) (Last *et al*. 2016).

As with other chondrichthyans, the most common fossil remains of batomorphs are their highly mineralized teeth and placoid scales. Based on such remains, it is possible to trace the fossil record of batomorphs back to the Early Jurassic (Pliensbachian; *c*. 185 Ma) of Germany (i.e. *Antiquaobatis* Stumpf & Kriwet, 2019) and the Toarcian (*c*. 180 Ma) of France, Belgium and Luxembourg (i.e. Toarcibatidae: *Toarcibatis* Delsate & Candoni, 2001, *Cristabatis* Delsate & Candoni, 2001, and *Doliobatis* Delsate & Candoni, 2001). The oldest holomorphic (i.e. more or less completely preserved) batomorph is known from the Toarcian of the Posidonia shale near Holzmaden, Germany (Maisey *et al*. 2020), which seems to have a somewhat different body shape than the Late Jurassic spathobatids. Its systematic affiliation remains unknown, but a pending study is likely to shed light on the evolution of primitive batomorphs.

The batomorph family Spathobatidae includes the genera *Asterodermus* (currently considered dubious), *Belemnobatis, Kimmerobatis* and *Spathobatis*. Spathobatid remains, consisting mainly of isolated teeth, are known from the Early Jurassic (Toarcian; Thies 1982) to the Early Cretaceous (Aptian, *c*. 118 Ma; Batchelor & Duffin 2023). Their geographical distribution comprises Asia (i.e. Thailand; Cuny *et al*. 2007) and Europe (i.e. France, e.g. Thiollière 1852; Delsate & Candoni 2001; Thies & Leidner 2011; Rees *et al*. 2013; Guinot *et al*. 2014; Germany, e.g. Agassiz 1836; Kriwet & Klug 2004; Thies & Leidner 2011; Kriwet & Klug 2015; Villalobos-Segura *et al*. 2023; the UK, e.g. Underwood & Rees 2002; Underwood & Ward 2004; Sweetman *et al*. 2014; Underwood & Claeson 2017; Batchelor & Duffin 2023; Penn & Sweetman 2023; Poland (Rees 2005); Switzerland (Leuzinger *et al*. 2017); and Spain, e.g. Kriwet *et al*. 2009).

Holomorphic spathobatids (i.e. *Asterodermus platypterus, Belemnobatis sismondae, Kimmerobatis etchesi* and *Spathobatis bugesiacus*) are known from the Late Jurassic, when batomorphs became more diverse and abundant (e.g. Cavin *et al*. 1995; Cione 1999; Arratia *et al*. 2002; Underwood & Ward 2004; Cuny *et al*. 2008, 2009). They are common in several Late Jurassic (Kimmeridgian–Tithonian, *c*. 156–145 Ma) fossil sites of Europe, which include the Solnhofen Archipelago, Germany (Kimmeridgian–Tithonian; e.g. Kriwet & Klug 2004, 2015; Villalobos-Segura *et al*. 2023), Cerin, France (late Kimmeridgian; e.g. Thiollière 1852), Kimmeridge, UK (early Tithonian; e.g. Underwood & Claeson 2017) and Canjuers, France (early Tithonian; e.g. Sauvage 1873; Cavin *et al*. 1995).

Since the first report of Late Jurassic articulated batomorphs from Germany in 1836 (Agassiz 1836; Münster 1836), several batomorph species have been described, many of which subsequently were synonymized with other taxa, are no longer valid, or have validity that is ambiguous and being debated. Probably the most controversial species to date is *Asterodermus platypterus* from the Upper Jurassic Solnhofen Archipelago, the holotype of which is incompletely preserved and lacks the entire head, which arguably is the most important region for species discrimination. This fact has led to taxonomic ambiguity, with different authors disagreeing as to whether both *Asterodermus platypterus* and *Spathobatis bugesiacus* occur in the Solnhofen Archipelago or whether only *As. platypterus* occurred there, while holomorphic specimens of *S. bugesiacus* are restricted to the French locality, Cerin (e.g. Frickhinger 1994, 1999; Kriwet & Klug 2015). Other authors, however, have proposed that *Asterodermus platypterus* should be treated as *nomen dubium* until diagnostic skeletal characters are recognized and described (Underwood & Rees 2002).

Here, we present a revision of Late Jurassic holomorphic batomorphs from Europe, using both qualitative and quantitative methodological approaches (i.e. geometric morphometrics) to quantify the differently shaped snouts of the batomorphs, and traditional morphometrics (i.e. body proportions). Our aim is to evaluate whether the currently accepted representation of batomorph species during the Late Jurassic is supported by ‘modern’ approaches, and whether these methods can provide further characters to identify these groups, the taxonomic identification of which has relied mostly on dental features so far.

## Material and Method

### Specimens

A total of 52 specimens of articulated Late Jurassic batomorphs were used in the present study (Table S1). The specimens are from upper Kimmeridgian (Cerin, France) and lower Tithonian (Solnhofen Archipelago, Germany; Kimmeridge, UK) deposits of Europe, and represent all hitherto known batomorph morphotypes of these fossil sites. Not available for this study, however, were *Spathobatis*? *morinicus* Sauvage, 1873 from the lower Tithonian of Canjuers, France, a holomorphic batomorph based on a single specimen (Sauvage 1873; Cavin *et al*. 1995), as well as an unnamed batomorph from the middle Tithonian of Argentina (Cione 1999). Published figures of these two specimens were not considered to be of high enough quality to be used for measurements.

All specimens were photographed with a digital camera positioned orthogonally to each specimen to avoid doubtful results due to a misaligned angle. Some specimens were examined under ultraviolet light following the technique described in Tischlinger & Arratia (2013) for better identification of specific skeletal structures.

*Institutional abbreviations*. AMNH, American Museum of Natural History, New York, NY, USA; BMMS, Bürgermeister-Müller-Museum, Solnhofen, Germany; CM, Carnegie Museum of Natural History, Pittsburgh, PA, USA; HMNS, Houston Museum of Natural Science, Houston, TX, USA; JME, Jura-Museum Eichstätt, Eichstätt, Germany; LF, Lauer Foundation, Wheaton, IL, USA; MB, Museum Bergér, Eichstätt, Germany; MCZ, Museum of Comparative Zoology, Cambridge, MA, USA; MDC, Musée des Confluences, Lyon, France; MGL, Musée cantonal de Géologie, Lausanne, Switzerland; MJML, Museum of Jurassic Marine Life, Kimmeridge, UK; MNB, Museum für Naturkunde, Berlin, Germany; MNHN, Muséum national d’Histoire naturelle, Paris, France; NHMUK, the Natural History Museum, London, UK; NRM, Naturhistoriska Riksmuseet, Stockholm, Sweden; RBINS, Royal Belgian Institute of Natural Sciences, Brussels, Belgium; SMNK, Staatliches Museum für Naturkunde Karlsruhe, Karlsruhe, Germany; SNSB-BSPG, Bayerische Staatssammlung für Paläontologie und Geologie, Munich, Germany; TM, Teylers Museum, Haarlem, Netherlands.

### Linear measurements

**Fig. 1:**
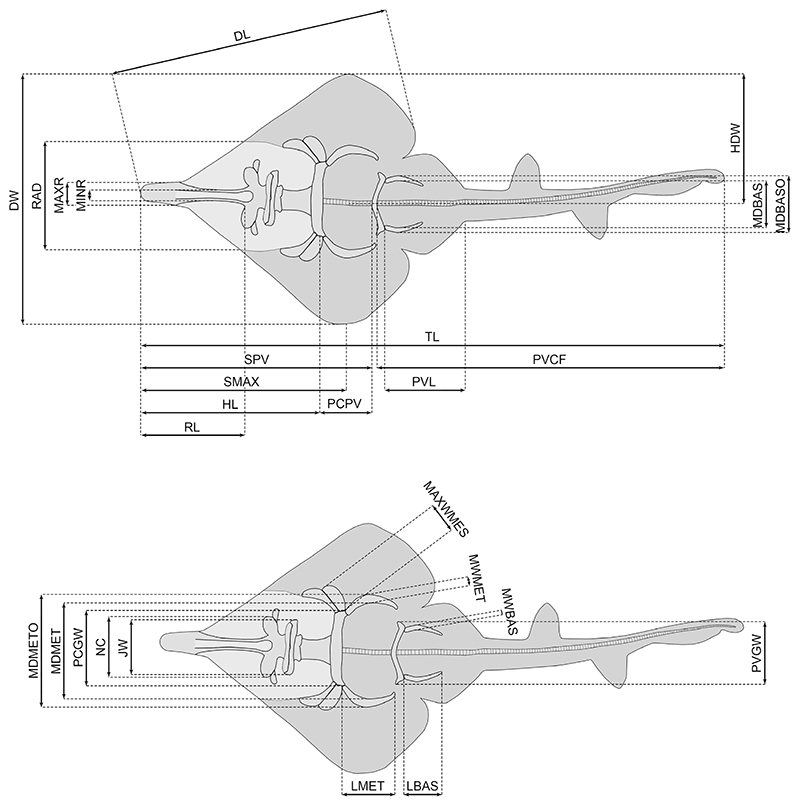
Schematic diagrams of the measurements used for traditional morphometric analyses. *Abbreviations*: DL, disc length; DW, disc width; HDW, half disc width; HL, head length; JW, jaw width; LBAS, length of basipterygia; LMET, length of metapterygia; MAXR, maximum rostrum width; MAXWMES, maximum width of mesopterygia; MDBAS, inner maximum distance between basipterygia; MDBASO, outer maximum distance between basipterygia; MDMET, inner maximum distance between metapterygia; MDMETO, outer maximum distance between metapterygia; MINR, minimum rostrum width; MWBAS, maximum width of basipterygia; MWMET, maximum width of metapterygia; NC, nasal capsules maximum width; PCGW, pectoral girdle width; PCPV, pectoral girdle to pelvic girdle; PVCF, pelvic girdle to caudal fin tip; PVGW, pelvic girdle width; PVL, pelvic fin length; RAD, span between anteriormost fin radials; RL, rostrum length; SMAX, distance from the tip of the snout to the point of maximum disc width; SPV, snout to pelvic girdle; TL, total length.

A total of 30 specimens were measured (Table S1), the remaining specimens were excluded due to their incomplete preservation. We used ImageJ (v1.53t) to take 27 different measurements (Fig. 1) on each specimen based on photographs to the nearest 0.001 mm. In total, each measurement was taken three times to avoid possible measurement errors. Subsequently, the mean value was used. A scale bar with 1 mm increments was included with all of the pictures, except for some of the pictures provided without scale bars by museum websites. In the pictures without a scale bar, pixels were measured with ImageJ because relative measurements rather than metric measurements were used later for the analyses; the taken measurements were adjusted as percentages of the total length (TL) of each individual for the species descriptions (Systematic Palaeontology, below) and as percentages of the disc width (DW) of each individual for statistical analysis.

The holotype of *Asterodermus platypterus* (NHMUK PV P 12067) lacks the head region and parts of the left pectoral fin. Given that the right pectoral fin is sufficiently complete to show the maximum disc width, it was mirrored and projected onto the left side. This enabled us to estimate the disc width of the holotype of *Asterodermus platypterus* and include the specimen in our analyses. Besides this, only specimens in which the pectoral fins were sufficiently preserved to identify the disc width were used. So far, no specimen of *Kimmerobatis etchesi* has been found with both or at least one complete pectoral fin preserved, thus we could not include *Kimmerobatis etchesi* in this analysis. Furthermore, it was not possible to obtain all measurements from some of the specimens included (e.g. due to incomplete preservation), resulting in our dataset also containing missing data. We first divided the dataset into *a priori* sorted subsets based on the initial qualitative classification of specimens to reduce the noise caused by the missing data in subsequent analyses, and imputed the missing values of each subset using a regularized iterative principal component analysis (PCA) algorithm with the function imputePCA in the R package missMDA (Josse & Husson 2016). The subsets with the implemented data were merged into a dataset containing all specimens and used in PCA.

To verify the *a priori* grouping of the specimens, we subsequently performed a linear discriminant analysis (LDA) using leave-one-out cross-validation (LOOCV; Table S2). For this, we used both continuous variables (i.e. measurements) and binary characters (absence/presence indicated by 0/1) that cannot be captured by measurements (i.e. direct scapulocoracoid–radial articulation, fin spines, antorbital cartilages, postpelvic processes, rostral appendix). All specimens were correctly assigned to the respective group (Table S2).

Several statistical tests were carried out to investigate differences between the groups. First, a Shapiro–Wilk test for normal distribution was applied to each measurement. Subsequently, we created subsets including either normally distributed or non-normally distributed relative measurements, respectively. The dataset containing non-normally distributed measurements was further examined with non-parametric tests. First, a Kruskal–Wallis test was applied to assess the differences of each measurement among groups. A pairwise Wilcoxon rank sum test with Bonferroni corrections for multiple comparisons for the differences between groups was conducted subsequently. The normally distributed measurements were examined for differences with an analysis of variance (anova) and a subsequent pairwise comparison considering the least squares means. Additionally, a second PCA was conducted with normally distributed measurements only.

All analyses were performed using R (v3.6.3; R Core Team 2013). Plots were created using the R packages ggplot2 (v3.4.0; Wickham 2016) and viridisLite (v0.4.1; Garnier 2018).

### Geometric morphometrics

**Fig. 2:**
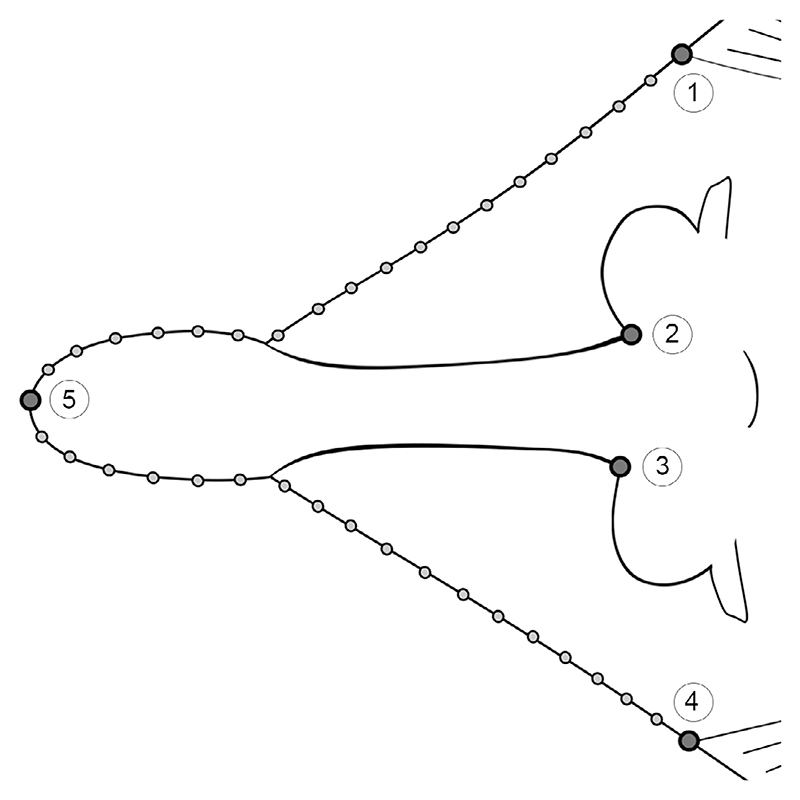
Location of the five true landmarks (large dots in dark grey) and 36 semilandmarks (small dots in light grey) for the geometric morphometric analyses. The true landmarks (1) and (4) are located at the point where the extension of the first radial of the propterygium reaches the edge of the disc, on the right and left pectoral fin respectively; (2) and (3) are located at the notches that indicate the connection between the base of the rostrum and the nasal capsules; (5) is located at the tip of the rostrum. The semilandmarks are arranged in two curves of 18 points each, one between landmarks (1) and (5) and one between landmarks (5) and (4). These two curves describe the shape of the head from the tip of the snout to the first pectoral radial.

The snout shape of 21 specimens (Table S1) was studied using 2D landmark-based geometric morphometrics. Five homologous landmarks were digitized using the software tpsDIG2 (v2.31; Rohlf 2017). Additionally, 36 semilandmarks were digitized between the homologous landmarks to capture the overall shape of the snout region (Fig. 2). A generalized Procrustes analysis (GPA) was performed on the landmark coordinates to minimize the variance caused by factors such as size, orientation, location and rotation (Rohlf & Slice 1990). The semilandmarks were allowed to slide using the minimum bending energy criterion (Bookstein 1997), given that it contributes to maintain the natural curvature and smoothness of the curves, as opposed to the Procrustes distance criterion, which can lead to excessive sliding of the semilandmarks and thus might cause artefacts and distortions in the analysed shape. To exclude the possibility of a Pinocchio effect (i.e. variation is limited to one or a few landmarks; e.g. Zelditch *et al*. 2012), we compared GPA and resistant fit theta-rho analysis (RFTRA) superimpositions with IMP CoordGen8 (Sheets 1998). The results of both superimposition methods indicate that no Pinocchio effect is present in our dataset (Fig. S1). Subsequently, the aligned coordinates underwent PCA to assess the shape variation of the snouts of the specimens. The only specimen of

*Kimmerobatis etchesi* in which the head region is preserved (i.e. MJML K874, holotype) was included in this analysis to identify its position in the PCA relative to the other groups; however, it was excluded from the following statistical analyses because multiple specimens of a group should be included to enable comparisons with other groups.

Following the PCA, we performed an LDA to verify the qualitative *a priori* classification of each specimen using an LOOCV. Out of 20 specimens, 19 were correctly assigned to the respective group (Table S3). The effect of size on shape as well as shape differences between the groups were estimated with a Procrustes anova with 1000 permutations, followed by pairwise comparisons between the groups, with the functions procD.lm and pairwise considering the distances between means in the R packages geomorph (v4.0.4; Adams *et al*. 2016) and RRPP (Collyer & Adams 2018), respectively. Additionally, an analysis of similarities (ANOSIM; Clarke 1993) was implemented to evaluate the extent of overlap between the recovered groups, as well as a permutational multivariate analysis of variance (PERMANOVA; Anderson 2001) to detect differences between group centroids. ANOSIM and PERMANOVA, each with 1000 permutations, were performed in PAST v2.17c (Hammer *et al*. 2001), Euclidean distances were chosen for distance measurement and the Bonferroni correction was used.

## Systematic Palaeontology

Regarding open nomenclature and rules for synonymy lists, we follow Matthews (1973), Bengtson (1988) and Sigovini *et al*. (2016). The selected synonym lists presented here cite publications in which articulated skeletal remains of the species in question were illustrated. Publications in which the species were only mentioned or described without an illustration are not cited, except in the case of descriptive literature prior to the official first description. In this case, the year of publication in italics means that the cited work mentions the species in question but does not contain a full description or illustration. An asterisk (*) preceding the year of publication indicates that the cited publication can be considered the official first description of the species in question, given that all of the necessary requirements according to the International Code of Zoological Nomenclature (ICZN) were met.

Class CHONDRICHTHYES Huxley, 1880
Subclass ELASMOBRANCHII Bonaparte, 1838
Cohort EUSELACHII Hay, 1902
Subcohort NEOSELACHII Compagno, 1977
Superorder BATOMORPHII Cappetta, 1980
Order Uncertain

*Remarks.* Late Jurassic holomorphic batomorphs show a surprising resemblance to modern Rhinopristiformes, especially to present-day guitarfishes (Rhinobatidae, Glaucostegidae) and wedgefishes (Rhinidae). Due to this superficial resemblance, they traditionally were assigned to the family Rhinobatidae (e.g. Zittel 1887–1890; Woodward 1889a, 1889b; Thies 1995; Underwood & Ward 2004; Kriwet *et al*. 2009; Thies & Leidner 2011; Klug & Kriwet 2013). However, recent phylogenetic studies based on morphological characters (Villalobos-Segura *et al*. 2019, 2022) and on a combination of molecular and morphological data (Jambura *et al*. 2023) posited that Late Jurassic batomorphs form a monophyletic group when maximum parsimony was used as optimality criterion. It should be noted that analyses of the same datasets with different optimality criteria (i.e. maximum likelihood and Bayesian inference) resulted in conflicting topologies and do not support a monophyletic group of Late Jurassic batomorphs (Villalobos-Segura & Underwood 2020, fig. 1; Villalobos-Segura *et al*. 2022, figs 50–51; Jambura *et al*. 2023, fig. S2). We follow the hypothesis common to these studies, which indicates that these batomorphs are sister to all remaining batomorphs and thus cannot be assigned to the family Rhinobatidae and therefore the order Rhinopristiformes. However, it currently also is not possible to identify an unambiguous monophyletic grouping for these batomorphs. Consequently, we temporarily place the Late Jurassic batomorphs in no order but classify them as *incerti ordinis*.

Family SPATHOBATIDAE Dames, 1888

*Nomenclatural remarks.* Currently, Underwood (2006) is considered to have introduced the family Spathobatidae for Jurassic batomorphs (Underwood & Claeson 2017). However, the reference to Underwood (2006) is incorrect for two reasons: first, Article 16.2. of the ICZN states that ‘a new family-group name published after 1999 must be accompanied by citation of the name of the type genus (i.e. the name from which the family-group name is formed)’, a criterion that Underwood (2006) does not meet. Second, the family name Spathobatidae was already established by Dames (1888), although this publication has been largely overlooked. Despite the fact that the name of the type genus is also not given here, this family is still valid because it was published before 1999; Article 11.7.1.1 of the Code states that ‘use of the stem alone in forming the name is accepted as evidence that the author used the generic name as valid in the new family-group taxon unless there is evidence to the contrary’, which is why we consider Dames (1888) as the correct reference for Spathobatidae.

Genus *Aellopobatis* nov.

*LSID.* https://zoobank.org/NomenclaturalActs/330C7704-2273-4756-A19A-A6C6BBF05F26

*Derivation of name.* Münster (1836) described a fossil consisting of a dorsal and a caudal fin, which may well be the first mention of this genus, albeit it is impossible to confirm due to the lack of diagnostic characters; he named it *Aellopos* after the harpy Aello, a hybrid in Greek mythology with the body of a bird and the head of a woman. To honour this original but preoccupied name, we complement it to *Aellopobatis*. The Greek *Ἀελλώ* (Aëllṓ) means ‘storm wind’, and *βατίς* (batís) means ‘ray’ or ‘skate’; feminine.

*Type species*. *Aellopobatis bavarica* sp. nov.

*Diagnosis.* As for type and only species.

*Stratigraphic & geographic distribution.* Upper Jurassic of Europe. Lower Tithonian of the Solnhofen Archipelago (Solnhofen, Eichstätt, Zandt, Kelheim, Langenaltheim, Blumenberg), Bavaria, Germany.

*Aellopobatis bavarica* sp. nov.

Figures 3, 4, S2

**Table:** 

1887–1890	*Spathobatis mirabilis* Wagner; Zittel, fig. 117.
1887	*Spathobatis mirabilis* Wagner; Zittel & Haushofer, palaeontological wall chart 34.
1889a	*Rhinobatus bugesiacus* (Thiollière); Woodward, fig. 3 [cop. Zittel, 1887-1890].
1889b	*Rhinobatus bugesiacus* (Thiollière); Woodward, text-fig. [cop. Zittel, 1887-1890].
1911	*Rhinobatus mirabilis* (Wagner); Zittel, fig. 115 [cop. Zittel, 1887-1890].
1914	*Rhinobatus bugesiacus* (Thiollière); Eastman, pl. 66, fig. 2.
1924	*Rhinobatus bugesiacus* (Thiollière); Abel, fig. 522 [cop. Zittel, 1887-1890].
1932	*Rhinobatus mirabilis* (Wagner); Zittel, fig. 125 [cop. Zittel, 1887-1890].
1991	*Aellopos bugesiacus* (Thiollière); Frickhinger, text-fig. [cop. Zittel, 1887-1890].
1994	*Aellopos bugesiacus* (Thiollière); Frickhinger, fig. 402.
1994	*Aellopos bugesiacus* (Thiollière); Barthel *et al.,* fig. 7.58.
1999	*Spathobatis bugesiacus* Thiollière; Frickhinger, figs 147-149.
1999	Indeterminated Ray; Frickhinger, fig. 150.
2004	*Asterodermus platypteros* Agassiz; Kriwet & Klug, fig. 18b, c.
2005	*Rhinobatos bugesiacus* (Thiollière); Vooren *et al.,* fig. 3.3 [cop. Zittel, 1887-1890].
2011	*Asterodermus* sp. Agassiz; Thies & Leidner, fig. 2.
2011	*Asterodermus* sp. Agassiz; Thies & Leidner, pl. 80, fig. A; pl. 84, fig. A.
2012	*Aellopos* Münster; Selden & Nudds, fig. 268.
2015	*Spathobatis bugesiacus* Thiollière; Kriwet & Klug, figs 685a, 685b, 687.
2015	*Spathobatis bugesiacus* Thiollière; Tischlinger, fig. 187.
2023	*Asterodermus platypterus* Agassiz; Villalobos-Segura *et al*., fig. 31c, e.
2023	*Spathobatis bugesiacus* Thiollière; Villalobos-Segura *et al*., fig. 32 (note that the scale in fig. 32C is incorrect).

*LSID.* https://zoobank.org/NomenclaturalActs/F0BDF3D2-D327-4536-B2AD-7E30A2165B95

*Derivation of name.* The species name *bavarica* is Latin and means ‘Bavarian’; feminine.

*Holotype.* SNSB-BSPG AS I 1377 (almost complete specimen lacking parts of the tail, female) and SNSB-BSPG AS I 1378 (almost complete specimen lacking parts of the tail, female); part and counterpart.

*Referred material.* SNSB-BSPG 1964 XXIII 577 (almost complete specimen, female); SNSB-BSPG AS VII 1170 (specimen has only parts of the caudal fin, the second dorsal fin and tail vertebrae preserved); SNSB-BSPG AS I 506 (complete specimen, male); SNSB-BSPG 1952 I 82 (almost complete specimen with damage on parts of the disc and the head region, female); SNSB-BSPG AS I 505 (complete specimen, female, complement to NHMUK PV P 6010); NHMUK PV P 6010 (complete specimen, female, complement to SNSB-BSPG AS I 505); CM 5396 (complete specimen, male); HMNS 686037 (complete specimen, female); SMNK-PAL 47090 (almost complete specimen lacking parts of the right pectoral and pelvic fins, female); MB 14-12-22-2 (counterplate preserved); MB 14-12-22-3 (complete specimen with small areas of damage on the rostrum, female); private collection of Helmut Tischlinger, specimen without number (complete specimen, female, figured in Tischlinger 2015, p. 113, fig. 187); LF 2323 (complete specimen, male); LF 205 (complete specimen, female); Tierpark + Fossilium Bochum, specimen without number (specimen lacking the rostrum and parts of the left pectoral fin, female); unknown private collection, specimen without number (complete specimen, male, figured in: Kriwet & Klug 2015, p. 347, fig. 687; Villalobos-Segura *et al*. 2023, p. 52, fig. 32A).

*Diagnosis.* Guitarfish-like batomorph that is unique in the combination of the following characters: exceptionally long rostrum with paddle-shaped rostral appendix; antorbital cartilages present but not extending halfway between the nasal capsules and the propterygium; 38–46 pectoral radials (9–11 propterygial, 9–12 mesopterygial and 20–23 metapterygial); no pectoral radials articulating directly with the scapulocoracoid between the meso- and metapterygium; pectoral radials segmented in up to five segments; at least 14 pairs of ribs; *c*. 21 basipterygial radials (including one compound radial); puboischiadic bar curved anteriorly; claspers long and slender; no fin spines present.

*Type locality.* Lower Tithonian; Kelheim, Bavaria, Germany.

### Description

*General body form.* Large-sized batomorph, reaching a total length of *c*. 170 cm. The body shape is guitarfish-like, with a strongly elongated rostrum. The disc is large, broad and heart-shaped, reaching an average width of 41.6% of the total length (range, 36.4–48.3% TL). With an average length of 48.6% TL, the disc is longer than wide (range, 44.5–54.3% TL). The tail is narrow and long and accounts for an average of 58.2% TL, measured from the pelvic girdle to the tip of the caudal fin (range, 52.3–61.2% TL). The two dorsal fins are located well behind the pectoral girdle, with the first dorsal fin originating at *c*. 63.3% TL (range, 60.1–65.7% TL), and the second dorsal fin originating at *c*. 73.9% TL (range, 72.3–75.5% TL). Both dorsal fins are nearly equal in size, the first dorsal fin has a height of *c*. 7.2% TL (range, 5.5–8.5% TL) and a width of *c*. 6.5% TL (range, 5.8–8.3% TL), the second dorsal fin has a height of *c*. 6.8% TL (range, 5.5–8.5% TL) and a width of *c*. 6.6% TL (range, 6.0–7.3% TL).

*Neurocranium.* The neurocranium is widest at the level of the nasal capsules, narrows at the orbits and widens again at the postorbital processes. Posterior to the short postorbital processes, the otic region does not narrow but gently widens towards the posterior articulation of the synarcual (e.g. CM 5396, SNSB-BSPG AS I 506, SMNK-PAL 47090).

The rostrum has a precerebral fossa dorsally and a mineralized rostral appendix that is paddle-shaped and strongly elongated. It narrows in the middle and is widest at the base at the nasal capsules and at the level of the rostral appendix. The rostrum is more than twice as long as the rest of the neurocranium. Measured from the tip of the rostrum to the level of the insertion point of the nasal capsules, it accounts for an average of 18.5% TL (range, 16.8–21.8% TL). The nasal capsules are elongated-oval and inclined anteriorly. They are approximately as wide as the jaw cartilages. The anterior margins of the nasal capsules are blunt; horn-like processes are absent. The antorbital cartilages are narrow but have a broad connection to the posterior part of the nasal capsules and are slightly posterolaterally directed. They are relatively short, reaching less than halfway between the nasal capsule and the propterygium. The supraorbital crest is weakly developed.

*Jaws & branchial skeleton.* Both jaw cartilages are relatively equal in anteroposterior depth. The anterolaterally directed dorsal flange of the Meckel’s cartilage articulates with the most lateral part of the palatoquadrate, which is best seen in ventrally exposed specimens.

The hyomandibulae are short, triangular and slightly curved anteriorly to connect to the jaw joint with a rounded, knob-like articulation. The articulation to the neurocranium is rather broad. The epibranchials and ceratobranchials are long and plate-like. The basihyal (visible in ventrally exposed specimens; e.g. HMNS 686037 or the privately owned specimen illustrated in Kriwet & Klug 2015, fig. 687 and Villalobos-Segura *et al*. 2023, fig. 32A) is rather long and narrow.

*Axial skeleton & unpaired fins.* The vertebral centra are cyclospondylic and are similar in size for a considerable part of the specimens, only in the second half of the tail do they slowly become smaller. The supraneural spines are well-developed and plate-like. At least 14 pairs of ribs are present in the pelvic region (Fig. S2C).

Almost the entire length of the synarcual contains fully developed vertebral centra. In dorsal view (e.g. SMNK-PAL 47090, SNSB-BSPG AS-I-505) the base of the lateral stays of the synarcual is clearly visible, giving the synarcual a trapezoidal shape. The lateral stays expand posteriorly and reach their maximum width at the level of the pectoral girdle. In ventral view (e.g. HMNS 686037 or the privately owned specimen illustrated in Kriwet & Klug 2015, fig. 687 and Villalobos-Segura *et al*. 2023, fig. 32A) it can be seen that the synarcual lip firmly fits into the notch in the basicranium.

The dorsal fins are large and of nearly equal size. No fin spines are associated with the dorsal fins.

*Pectoral girdle & fins.* The scapular processes (e.g. MB 14-12-22-2) are rather narrow and taper towards the apex (Fig. S2A). The coracoid bar (best seen in ventrally exposed specimens such as LF 2323, HMNS 686037, or the privately owned specimen illustrated in Kriwet and Klug 2015, fig. 687 and Villalobos-Segura *et al*. 2023, fig. 32A) is broad and straight, tapering on both sides towards the fusion points with the scapular processes, which extend dorsally (Fig. S2B). The three basal cartilages directly articulate with the scapulocoracoid (Fig. S2A). The anteriorly elongated propterygium is paddle-shaped and associated with 9–11 radials. The mesopterygium is somewhat oval, directed anteriorly and tangent to the propterygium; 9–12 radials articulate with the mesopterygium. The metapterygium is long and curved and typically articulates with 20–23 radials. None of the radials articulates directly with the scapulocoracoid between the meso- and metapterygium. The radials typically have five segments.

*Pelvic girdle, fins & claspers.* The puboischiadic bar is curved anteriorly and has lateral prepelvic processes. The basipterygium is elongated and slender and only slightly curved inwards. The bar-shaped anterior compound radial is directed posterolaterally and is followed by *c*. 20 radials (Fig. S2C). The tail (pelvic girdle to caudal fin tip) is long, accounting for an average of 59.2% of the total length (range, 52.3–61.2% TL).

CM 5396, SNSB-BSPG AS I 506, LF 2323 and the privately owned specimen illustrated in Kriwet & Klug (2015, fig. 687) and Villalobos-Segura *et al*. (2023, fig. 32A) are adult male specimens, with relatively long and slender claspers. The basal elements (indeterminate elements and β-cartilage) can be approximated but are difficult to distinguish from each other. From the axial elements (ventral marginal, dorsal marginal and axial cartilages) it appears that the axial cartilage is slightly rugose, but not as pronounced as in *Kimmerobatis etchesi* (Underwood & Claeson 2017). Adjacent to the axial cartilage is a long and smooth dorsal marginal cartilage (e.g. Kriwet & Klug 2015, fig. 687; Villalobos-Segura *et al*. 2023, fig. 32A) and ventral marginal cartilage (e.g. LF 2323). The last elements of the claspers are composed of the terminal cartilages. The dorsal terminal 1 cartilage is oval in shape (Fig. S2D; Kriwet & Klug 2015, fig. 687; Villalobos-Segura *et al*. 2023, fig. 32A).

*Teeth & denticles.* The tooth morphology is known from Thies & Leidner (2011), who extracted teeth from SNSB-BSPG AS I 1377 and SNSB-BSPG 1964 XXIII 577 (both at that time described as *Asterodermus* sp.). The anterior teeth are mesiodistally expanded, with the labial face being *c*. 2.5–3-fold wider than high. The median cusp is only rudimentarily developed, the labial apron is not separated from the crown and is indicated only by the rounded outline of the labial tooth face. The lingual uvula is narrow but well-developed and bulbous. A mesiodistal ridge separates the labial and lingual faces of the crown. The root is hemiaulacorhize. Lateral teeth are nearly identical in size and proportions to anterior teeth, but both hemi- and holaulacorhize root vascularization patterns are developed.

Dermal denticles are of the same morphotype as in other spathobatids, with a star-shaped base and a small, knob-like crown, often arrow-shaped.

### Comparison

Large-sized guitarfish-like batomorph reaching a total length of *c*. 170 cm, differentiating it from *As. platypterus, B. sismondae* and *S. bugesiacus*, which reach a significantly smaller total length as adults (the comparison with *K. etchesi* is not possible at this point because no completely preserved specimen has yet been found, however, the posterior section of a mature male specimen is smaller than that of similarly mature *A. bavarica*). Further morphological characters of *A. bavarica* include: rostral appendix present (unlike *B. sismondae* and *K. etchesi*, but like *As. platypterus* and *S. bugesiacus*, albeit apparently much more pronounced); antorbital cartilages present (unlike *B. sismondae*), but not extending halfway between the nasal capsules and the propterygium (unlike *As. platypterus*, but like *S. bugesiacus* and *K. etchesi*); no pectoral radials are directly articulating with the scapulocoracoid between the meso- and metapterygium (unlike *S. bugesiacus*, but like *As. platypterus, B. sismondae* and *K. etchesi*); pectoral radials segmented in up to five segments (unlike *As. platypterus*, but like *S. bugesiacus*); puboischiadic bar curved anteriorly (unlike *As. platypterus* and *B. sismondae*, but like *S. bugesiacus*); no fin spines present (unlike *As. platypterus, S. bugesiacus* and *B. sismondae*, but like *K. etchesi*).

### Nomenclatural remarks

Fragmentary remains of a large batomorph that might belong to *Aellopobatis bavarica* were found in the Kelheim region of the Franconian Alb, Bavaria, and described by Münster (1836) as ‘*Aellopos*’ *elongata*. It is possible that this short description is based on SNSB-BSPG AS VII 1170, a fossil consisting only of a partly preserved tail and therefore lacking diagnostic characters. However, Münster (1836) was apparently unaware of an earlier described lepidopteran genus, *Aellopos* Huebner, 1819, rendering the generic name unavailable. Agassiz (1843, p. 382) introduced *Euryarthra münsteri* based on a large pectoral fin from the Solnhofen area and noted that the associated batomorph ‘must have been one of the largest in the family’. Later, Wagner (1857) provided a brief description of *Spathobatis mirabilis* based on a large, almost complete batomorph specimen from the Solnhofen area. Subsequently, Wagner (1862) provided a more detailed description, in which he also described the absence of a pectoral fin, but without providing an illustration of the specimen. Although he noted a strong resemblance between *S. mirabilis* and the smaller *Spathobatis bugesiacus*, which had been described by Thiollière (1852) from the deposits of Cerin, he stated that the large difference in size justified the assignment of his specimen as a new species (Wagner 1857, 1862). Eventually, Zittel (1887–1890) illustrated *S. mirabilis* and also included ‘*Aellopos*’ and *Euryarthra* in the genus *Spathobatis*. The illustrated batomorph, however, does not represent the same specimen described by Wagner (1857, 1862), given that both pectoral fins are present. Woodward (1889a, 1889b) subsequently merged ‘*Aellopos*’, *Euryarthra* and *Spathobatis* within *Rhinobatus*, stating that there was no difference between the extant and extinct taxa to justify a separate genus. Furthermore, he merged *S. mirabilis* together with *S. bugesiacus* into *Rhinobatus bugesiacus*, because he believed that the difference in size between the German and French specimens was no reason to justify separate species (Woodward 1889a, 1889b). The merging of the two taxa has persisted since then, albeit mostly under the name *Spathobatis bugesiacus*.

Although the specimen illustrated by Zittel (1887–1890), at the time stored in the Bayerische Staatssammlung für Paläontologie und Geologie München, was unfortunately destroyed during World War II, a cast of the specimen is still on display to this day in the American Museum of Natural History in New York (cast 7494). Furthermore, the counter plate of the destroyed specimen is currently on display in the Carnegie Museum of Natural History (CM) in Pittsburgh, USA (CM 5396).

The specimen that was described by Wagner (1857, 1862), but never illustrated, was most probably lost during World War II and we therefore cannot be sure that the specimen illustrated by Zittel (1887–1890) represents the same species. Ultimately, this requires us to consider ‘*Aellopos’ elongata* as invalid and we therefore introduce a new species named *Aellopobatis bavarica* to avoid possible taxonomic conflict.

Genus *Asterodermus* Agassiz, 1836

*Type species. Asterodermus platypterus* Agassiz, 1836.

*Derivation of name.* The genus name *Asterodermus* is composed of the Greek words *ἀατήρ* (astḗr), meaning ‘star’ and *δέρμα* (dérma), meaning ‘skin’, due to the star-shaped dermal denticles covering the body.

*Stratigraphic & geographic distribution.* Upper Jurassic of Europe. Lower Tithonian of the Solnhofen Archipelago (Solnhofen, Eichstätt, Kelheim, Blumenberg, Birkhof), Bavaria, Germany.

*Asterodermus platypterus* Agassiz, 1836

Figure 5

**Table:** 

1836	*Asterodermus platypterus* Agassiz, p. 381.
* 1843	*Asterodermus platypterus* Agassiz, pl. 44, fig. 2.
1850–1856	*Asterodermus platypterus* Agassiz; Bronn & Roemer, pl. 25, fig. 5.
1859	*Asterodermus platypterus* Agassiz; Meyer, pl. 1, fig. 1.
1914	*Belemnobatis sismondae* Thiollière; Eastman, pl. 67, fig. 1.
2000	*Asterodermus platypterus* Agassiz; Viohl, fig. 7.
2004	*Asterodermus platypteros* Agassiz; Kriwet & Klug, figs 18a, 19.
2011	*Asterodermus platypterus* Agassiz; Thies & Leidner, pl. 70.
2011	† *Spathobatis* Thiollière; Claeson & Hilger, fig. 6D–E.
2011	*Spathobatis bugesiacus* Thiollière; Thies & Leidner, pl. 99.
2015	*Spathobatis bugesiacus* Thiollière; Rettondini Laurini, fig. 34a.
2015	*Asterodermus platypterus* Agassiz; Rettondini Laurini, fig. 34f.
2015	*Spathobatis bugesiacus* Thiollière; Kriwet & Klug, fig. 685c.
2015	*Asterodermus platypterus* Agassiz; Kriwet & Klug, fig. 686.
2017	*Asterodermus platypterus* Agassiz; Moser, fig. 24.
2019	*Spathobatis bugesiacus* Thiollière; Villalobos-Segura *et al*., figs S.1.B., S.5.D.
2020	*Spathobatis bugesiacus* Thiolliere; Villalobos-Segura & Underwood, figs S1B, S4D.
2023	*Asterodermus platypterus* Agassiz; Villalobos-Segura et al., fig. 31a, b, d.

*Derivation of name.* The species name *platypterus* is composed by the prefix *platy*-, derived from the Greek *πλατύς* (platús), meaning ‘flat’ or ‘wide’, and *πτερόν* (pterón), meaning ‘wing’; masculine.

*Holotype.* NHMUK PV P 12067 (incomplete specimen lacking the head and parts of the left pectoral fin, female).

*Referred material.* NHMUK PV P 10934 (complete specimen, female); JME SOS-2212a (almost complete specimen lacking the tip of the rostrum and the tip of the tail, female); JME SOS-3647 (almost complete specimen lacking the tip of the tail, female); SNSB-BSPG AS XIX 502 (complete specimen consisting of plate and counter plate, female); CM 4408 (complete specimen, female).

*Diagnosis.* Guitarfish-like batomorph; unique in the combination of the following characters: knob-like rostral appendix present; antorbital cartilages present and extending up to halfway between the nasal capsules and the propterygium; 39–47 pectoral radials (8–10 propterygial, 9–12 mesopterygial, 22–25 metapterygial); no pectoral radials directly articulating with the scapulocoracoid between the meso- and metapterygium; pectoral radials segmented in up to four segments; at least eight pairs of ribs; *c*. 19 basipterygial radials (including one compound radial); pelvic girdle almost straight; presence of minute fin spines supporting both dorsal fins.

*Type locality.* Lower Tithonian; Kelheim, Bavaria, Germany.

### Description

*General body form.* Medium-sized batomorph, reaching a total body length of *c*. 60 cm. The disc is more or less wedge-shaped, reaching a width of *c*. 40.5% of the total length (range, 32–48.2% TL). With an average length of 43.9% TL (range, 36.6–52% TL), the disc is longer than it is wide.

*Neurocranium.* The neurocranium reaches its widest point at the level of the nasal capsules, narrows at the orbits and widens again at the postorbital processes. Posterior to the postorbital processes, the otic region becomes narrower towards the posterior articulation of the synarcual. In dorsal view the rostrum has a precerebral fossa and a mineralized rostral appendix in the shape of a small knob. The rostrum is longer than the rest of the neurocranium. The rostrum is of moderate length compared with other spathobatids and accounts for an average of 13.9% TL (range, 11.6–15.8% TL). It narrows in the middle and is widest at the base of the nasal capsules and at the tip of the snout.

The nasal capsules are elongated-oval and inclined anteriorly. They are as wide as the jaw cartilages. The anterior margins of the nasal capsules are blunt; horn-like processes are absent. The antorbital cartilages are elongated and narrow and are connected to the posterior part of the nasal capsules and are directed slightly posterolaterally. There is no connection between the pectoral fins and the antorbital cartilages, as the latter only extend up to halfway from the nasal capsules to the propterygium. The supraorbital crest is weakly developed. Short postorbital processes are present.

*Jaws & branchial skeleton.* The palatoquadrate and Meckel’s cartilage are relatively equal in anteroposterior depth. An anterolaterally directed dorsal flange is present on the Meckel’s cartilage, articulating with the palatoquadrate. The hyomandibulae are broad at the base where they are connected to the neurocranium, but at the distal end they are directed anterolaterally and form a kind of narrow hook with which they are connected to the jaw joint. The epibranchials and ceratobranchials are plate-like.

*Axial skeleton & unpaired fins.* Fully developed vertebral centra are present along almost the entire length of the synarcual. Supraneural spines are elongated and plate-like and are present from the pectoral girdle onwards. At least eight pairs of ribs at the level of the pelvic girdle are present. Minute fin spines are present in front of both dorsal fins.

*Pectoral girdle & fins.* The pectoral girdle is directly articulating with the basal cartilages, with the articulation of the mesopterygium being the longest. The propterygium is crescent-shaped and narrow. The mesopterygium is somewhat oval, directed anteriorly, and reaches about two-thirds the length of the propterygium. The metapterygium is long, narrow and curved. A total of 8–10 radials articulate with the propterygium, 9–12 with the metapterygium, and 22–25 with the mesopterygium. None of the radials articulates directly with the scapulocoracoid between the meta- and mesopterygium. The radials are typically four-segmented, with the distalmost segment being bifurcated.

*Pelvic girdle, fins & claspers.* The pelvic girdle is straight and has lateral prepelvic processes. The basipterygium is elongated and slender and only slightly curved inwards. The bar-shaped anterior compound radial is directed posterolaterally and is followed by *c*. 18 radials. The tail length (measured from pelvic girdle to caudal fin tip) averages 53.8% TL (range, 49.8–58.4% TL).

No specimens with claspers (i.e. male sex) could be identified during this study.

*Teeth & denticles.* The tooth morphology in *Asterodermus platypterus* was examined by Thies & Leidner (2011), who removed teeth of specimen NHMUK PV P 10934 (at that time described as *S. bugesiacus*). The described teeth are from unknown positions in the jaw. They are mesiodistally expanded. The crown surface is smooth and the labial surface is *c*. 2.5-fold as wide as it is high. A mesiodistal ridge extends over the entire crown, separating the labial and lingual face. A median cusp is absent or only rudimentary developed. The apron is small, peg-like and detached from the crown, conversely to the condition in *Aellopobatis bavarica*, in which the apron is either completely reduced or at least (if present) not detached from the crown. The uvula is well-developed and bulbous. The root is holaulacorhize.

The basal surface of the dermal denticles is typically flat and star-shaped. The crowns are smooth and either bulbous or thorn-like and elongated (Thies & Leidner 2011).

### Comparison

A knob-like rostral appendix is present (unlike *B. sismondae* and *K. etchesi*, but similar to *S. bugesiacus* and *A. bavarica*); antorbital cartilages present (unlike *B. sismondae*) and extending up to halfway between the nasal capsules and the propterygium (unlike *A. bavarica, K. etchesi* and *S. bugesiacus*); no pectoral radials directly articulating with the scapulocoracoid between the meso- and metapterygium (unlike *S. bugesiacus*, but like *A. bavarica, B. sismondae* and *K. etchesi*); pectoral radials segmented in up to four segments (unlike *A. bavarica* and *S. bugesiacus*); pelvic girdle almost straight (unlike *A. bavarica* and *S. bugesiacus* but similar to *B. sismondae*); presence of minute fin spines supporting both dorsal fins (unlike *A. bavarica* and *K. etchesi*, but like *S. bugesiacus* and *B. sismondae*, albeit the spines are markedly larger in the latter species).

Genus *Belemnobatis* Thiollière, 1852

*Type species. Belemnobatis sismondae* Thiolliére, 1852.

*Derivation of name.* The genus name *Belemnobatis* is derived from the Greek words *βέλεμνoν* (bélemnon), meaning ‘spear’, probably because of the large fin spines, and *βατίς* (batís), meaning ‘ray’ or ‘skate’; feminine.

*Stratigraphic & geographic distribution.* Upper Jurassic of Europe. Upper Kimmeridgian of Cerin, Ain, France.

*Belemnobatis sismondae* Thiollière, 1852

Figure 6

**Table:** 

1852	*Belemnobatis sismondae* Thiollière, pl. 8, fig. 1.
1854–1873	*Belemnobatis sismondae* Thiollière, pl. 3, fig. 1.
1949	*Belemnobatis sismondae* Thiollière; de Saint-Seine, pls 3B, 4.
1991	*Belemnobatis sismondae* Thiollière; Frickhinger, p. 198.
2011	*Belemnobatis sismondae* Thiollière; Thies & Leidner, pls 87–92.

*Derivation of name.* The species name *sismondae* is dedicated to Angelo Sismonda, an Italian geologist.

*Holotype.* MDC 20015305 (almost complete specimen lacking a part of the tail, female).

*Referred material.* MDC 20015753 (almost complete specimen lacking parts of the left pectoral fin, male); MDC 20015318 (incomplete specimen with only pelvic fins, claspers, tail vertebrae, and fin spines preserved, male); MDC 20015262 (complete specimen, female); MDC 20015263 (complete specimen, female); MDC 20015265 (partly preserved specimen lacking the caudal region, sex unknown due to preservation); MDC 20015302 (partly preserved specimen showing the jaws and the branchial region, the pectoral girdle with the basal cartilages, and the pelvic girdle, sex unknown due to poor preservation); MDC 20015309 (complete specimen, female); MDC 20015310 (neurocranium); MDC 20015264 (incomplete specimen showing parts of the cranial and pectoral region); MDC 20015312 (part of the RBINS collection of the Musée des Confluences Lyon, currently on exhibit in the Evolution Gallery of the Royal Belgian Institute of Natural Sciences Brussels, complete specimen, female); MGL 38-774 (complete specimen, male); NRM P 1569 (almost complete specimen, lacking parts of the right pectoral fin and the tail, female); MNHN CRN-13 (partly preserved specimen showing the cephalic region and parts of the pectoral fin, sex unknown due to preservation); MNHN CRN-68 (partly preserved specimen with the cephalic region and parts of the pectoral fin, sex unknown due to preservation).

*Diagnosis.* Guitarfish-like batomorph characterized by a unique combination of the following characters: almost round disc that is about as long as wide; blunt snout; no rostral appendix present; no antorbital cartilages are connected to the nasal capsules; 40–43 pectoral radials (9–10 propterygial, 10–11 mesopterygial, and 21–22 metapterygial); all pectoral radials articulating with the basal cartilages, none is directly connected to the scapulocoracoid; pelvic girdle is rather straight; claspers are long and narrow; dorsal fins are supported by fin spines, which are fairly large and conspicuous.

*Type locality.* Upper Kimmeridgian; Cerin, Ain, France.

### Description

*General body form*. Medium-sized guitarfish-like batomorph, reaching a total length of *c*. 105 cm. The pectoral fins are fused at the cephalic portion of the body, forming an almost round, large disc that is about as long as it is wide. It reaches a width of *c*. 45.4% of the total length (range, 43.3–48.4% TL) and a length of *c*. 45% TL (range, 40.1–51.5% TL).

*Neurocranium.* The neurocranium is a box-like structure that appears to have the characteristic bottle shape of other batomorphs, reaching its maximum width at the nasal capsules, narrowing at the orbits, and widening again at the level of the postorbital processes and otic region. The rostral cartilage is short, accounting for an average of 8.5% TL (range, 6.7–9.9% TL), and supports a rounded and blunt snout. The rostrum is slightly longer than half of the length of the neurocranium and remains the same width over its entire length. In dorsal view the precerebral fossa is clearly discernible. No rostral appendix is present on the anteriormost portion of the rostral cartilage. The rostral cartilage is connected to the nasal capsules posteriorly, which are laterally extended and have a straight anterior surface without horn-like processes. No preorbital processes are discernible in the few specimens preserved in dorsal view (e.g. MDC 20015310). There is no evidence of antorbital cartilages attached to the posterolateral surface of the nasal capsules in the specimens examined.

*Jaws & branchial skeleton.* The jaw cartilages are similar in anteroposterior depth. The ceratobranchials are long and plate-like. A well-developed basihyal, which is narrow and longer than wide, is present behind the neurocranium (e.g. NRM P 1569).

*Axial skeleton & unpaired fins.* Dorsally and ventrally, the vertebral centra seem to have extended well into the midlength of the synarcual (e.g. MNHN CRN-13, MNHN CRN-68, NRM P 1569). No synarcual medial crest was observed in the specimens revised, but a synarcual lip that fits well into the notch of the basicranium, is present. The vertebral centra are cyclospondylic and similar in size over most of the specimens, gradually becoming smaller only in the second half of the tail. The supraneural spines are well-developed and plate-like. Several pairs of ribs are present, however, it was not possible to determine a number due to the state of preservation of the examined specimens. Two large fin spines are associated with the dorsal fins, noticeably larger than those of other spathobatids (i.e. *As. platypterus* and *S. bugesiacus*).

*Pectoral girdle & fins.* The coracoid is broad and straight. The scapular processes are broad at the connection point with the coracoid, but taper towards the apex. There are 40–43 pectoral radials; all radials articulate directly with the three basal cartilages. Nine to 10 radials are attached to the crescent-shaped propterygium. The mesopterygium is narrow and elongate, directed anteriorly, and tangent to the propterygium along its entire length. Ten to 11 radials are attached to the mesopterygium. The metapterygium is narrow and curved and associated with 21–22 radials.

*Pelvic girdle, fins & claspers.* The puboischiadic bar (i.e. pelvic girdle) is slightly curved and has distinct lateral prepelvic processes. The basipterygium is narrow and almost straight. The caudal peduncle is long, the tail length (pelvic girdle to caudal fin tip) is *c*. 60.3% TL (range, 53.8–64.7% TL).

Specimens MGL 38-774 and MDC 20015753 are adult male individuals of *c*. 93 cm and 104 cm total length, respectively, with long and well-developed claspers. They are narrow and possess long dorsal and ventral marginal cartilages adjacent to the axial cartilage. The terminal portion of the claspers is oval in shape.

*Teeth & denticles.* Teeth of holomorphic specimens were described by de Saint-Seine (1949) and have a somewhat rhombus shaped, transversely expanded labial face lacking a distinct labial apron. A fine mesiodistal ridge separates the labial and lingual faces. A median cusp is not or only slightly developed. The lingual uvula is straight and narrow. A deep nutritive groove is present in the root.

The dermal denticles have thickened, smooth, knob-like crowns. The base is larger than the crown, and the basal surface is flat and star-shaped, similar to that in *As. platypterus* (Thies & Leidner 2011). Enlarged dermal denticles are distributed throughout the body, particularly at the midline of the body.

### Comparison

Blunt snout (unlike *A. bavarica, As. platypterus, K. etchesi* and *S. bugesiacus*); no rostral appendix present (unlike *A. bavarica, As. platypterus* and *S. bugesiacus*, but similar to *K. etchesi*); no antorbital cartilages are present (unlike *A. bavarica, As. platypterus, K. etchesi* and *S. bugesiacus*); all pectoral radials articulate with the basal cartilages, none is directly connected to the scapulocoracoid (unlike *S. bugesiacus* but similar to *A. bavarica, As. platypterus* and *K. etchesi*); pelvic girdle is rather straight (unlike *A. bavarica* and *S. bugesiacus* but similar to *As. platypterus*); the dorsal fins are associated with fin spines (unlike *A. bavarica* and *K. etchesi* but like *As. platypterus* and *S. bugesiacus*), which are fairly large and conspicuous (unlike *As. platypterus* and *S. bugesiacus*).

Genus *Kimmerobatis* Underwood & Claeson, 2017

*Type species.* *Kimmerobatis etchesi* Underwood & Claeson, 2017.

*Derivation of name.* The genus name *Kimmerobatis* is derived from the type locality at Dorset (UK) and the Greek word *βατίς* (batís), meaning ‘ray’ or ‘skate’; feminine.

*Stratigraphic & geographic distribution.* Upper Jurassic of Europe. Lower Tithonian of the Upper Kimmeridge Clay Formation, East of Kimmeridge Bay, Dorset, southern England.

*Kimmerobatis etchesi* Underwood & Claeson, 2017

Figure 7

**Table:** 

2010	Rhinobatidae Etches & Clarke, p. 218, pl. 72.
* 2017	*Kimmerobatis etchesi* Underwood & Claeson, figs 1–3.
2020	*Kimmerobatis etchesi* Underwood & Claeson; Underwood, text-fig. 2.6E-F.

*Derivation of name.* The species name *etchesi* is derived from the name of the collector and preparator of the holotype and paratype, Steve Etches.

*Holotype.* MJML K874 (incompletely preserved specimen in dorsal view, lacking the tail and parts of the fins, female).

*Referred material.* MJML K1894 (paratype, incomplete specimen comprising parts of the pelvic girdle and one pelvic fin, both claspers, and parts of the caudal vertebrae, male).

*Diagnosis.* Guitarfish-like batomorph characterized by a unique combination of the following characters: pointed snout lacking a rostral appendix; antorbital cartilages present but reaching less than halfway between the nasal capsules and the unsegmented propterygium; no pectoral radials are directly articulating with the scapulocoracoid between the meso- and metapterygium; broad caudal region in proportion to disc; vertebra of caudal region shorter than neural spines; claspers long, narrow, and exhibiting rugose axial cartilages; presence or absence of fin spines not evident.

*Type locality.* Lower Tithonian: Upper Kimmeridge Clay Formation, East of Kimmeridge Bay, Dorset, UK.

### Description

*General body form.* The complete body outline remains unknown due to preservation (note that some of the body outline of the holotype MJML K874 is reconstructed); it is apparent, however, that the pectoral fins are fused to the pointed rostrum, forming a wedge-shaped disc that is slightly longer than wide. The well-developed caudal peduncle is relatively thick and at least as wide as the broadest part of the neurocranium.

*Neurocranium.* The rostral cartilage is robust and shows a prominent precerebral fossa in dorsal view. Only parts of the rostrum are preserved but it seems to be longer than the rest of the neurocranium. It is likely that a mineralized rostral appendix is absent, but its absence or presence cannot be unequivocally determined due to taphonomic processes. The nasal capsules are oval, inclined anteriorly, and have a smooth surface that lacks horn-like processes. The antorbital cartilages are relatively broad and triangular; they are connected to the posterolateral part of the nasal capsules, and reach less than half the distance between the nasal capsules and the propterygium. A weak supraorbital crest is present above the orbital area. Short postorbital processes are present. The neurocranium widens slightly in the otic region towards the articulation with the hyomandibulae and synarcual.

*Jaws & branchial skeleton.* Both Meckel’s cartilage and palato-quadrate are relatively robust and about the same anteroposterior depth. An anterolaterally directed, hook-like dorsal flange of the Meckel’s cartilage articulates with the palatoquadrate. The hyomandibulae are short, triangular and plate-like.

*Axial skeleton & unpaired fins.* The synarcual comprises large and fully formed centra for most of its length. A narrow medial crest is present on the synarcual. The lateral stays start to expand at about half of the length of the synarcual; they reach their maximum width at around two-thirds of the entire length of the synarcual. The vertebral centra are cyclospondylic and remain similar in size along the entire preserved body of MJML K874. The neural spines of the caudal region are longer than the respective vertebral centra. At least 19 pairs of ribs are present at the level of the pelvic girdle.

*Pectoral girdle & fins.* All pectoral radials articulate directly with the three basal pectoral elements. Seven radials articulate with the first element of the broad and unsegmented propterygium. The mesopterygium is long and tangential to the propterygium. The metapterygium is long, crescent-shaped, and associated with *c*. 22 radials.

*Pelvic girdle, fins & claspers.* The puboischiadic bar is straight, the basipterygium is narrow and gently curved. About 21 radials are articulated with the basipterygium.

The claspers, as seen in MJML K1894, are long and slender and are composed of rugose axial cartilage. Long and smooth marginal cartilages are present, tangential to the axial cartilages. The terminal elements of the claspers are irregularly oval shaped.

*Teeth & denticles.* Detailed studies on the tooth and placoid scale morphology of *Kimmerobatis etchesi* are still pending. There is no evidence for the presence of star-shaped denticles. Teeth have not been extracted from the holotype, but are clearly small in relation to the jaw size relative to other genera. They are rather equant and less laterally expanded than those of *A. bavarica, As. platypterus* and *B. sismondae*.

### Comparison

Pointed snout, neither blunt nor with a knob-like or paddle-shaped projection (unlike *A. bavarica, As. platypterus, B. sismondae* and *S. bugesiacus*); antorbital cartilages present (unlike *B. sismondae*) but reaching less than halfway between the nasal capsules and the propterygium (unlike *As. platypterus* but similar to *A. bavarica* and *S. bugesiacus*); no pectoral radials are directly articulating with the scapulocoracoid between the meso- and metapterygium (unlike *S. bugesiacus* but like *A. bavarica, As. platypterus* and *B. sismondae*).

Genus *Spathobatis* Thiollière, 1852

*Type species*. *Spathobatis bugesiacus* Thiolliére, 1852.

*Derivation of name.* The genus name *Spathobatis* is derived from the Greek *σπᾰ́θη* (spáthē), which means ‘blade’, probably because of the elongated rostrum, and *βατίς* (batís), meaning ‘ray’ or ‘skate’; feminine.

*Stratigraphic & geographic distribution.* Upper Jurassic of Europe. Upper Kimmeridgian of Cerin, Ain, France.

*Spathobatis bugesiacus* Thiollière, 1852

Figure 8

**Table:** 

1849	*Spathobatis bugesiacus* Thiollière.
* 1852	*Spathobatis bugesiacus* Thiollière, pl. 9.
1894	*Rhinobatus bugesiacus* (Thiollière); Jaekel, fig. 1
1949	*Spathobatis bugesiacus* Thiollière; de Saint-Seine, pls 1B, 2, 3A & C.
1988	*Spathobatis* Thiollière; Carroll, fig. 5.16b.
1993	*Spathobatis* Thiollière; Carroll, fig. 5.17b.
2011	*Spathobatis bugesiacus* Thiollière; Thies & Leidner, pls 93, 94, 96.
2014	*Spathobatis bugesiacus* Thiollière; Bernier *et al.,* fig. 9F.
2015	*Spathobatis bugesiacus* Thiollière; Bernier & Gaillard, figs 1048, 1059.

*Derivation of name.* The species name *bugesiacus* is derived from the historical region ‘Bugey’ in France, the location of Lagerstätte Cerin, where the holotype of *Spathobatis bugesiacus* was found.

*Holotype.* MDC 20015307 (almost complete specimen lacking the tail, female).

*Referred material.* MDC 20015303 (complete specimen, female); MDC 20015301 (specimen lacking parts of its disc and tail, female); MDC 20015298 (specimen lacking the tail and parts of the disc, male); MDC 20015304 (incomplete specimen lacking parts of its cranial and caudal region, female, plate and counterplate preserved); MDC 20015308 (specimen lacking parts of the rostrum, parts of the disc, and the tail, female); MDC 20150396 (imprint of a partly preserved specimen lacking parts of its disc and tail, female); NHMUK PV P 2099 (specimen lacking parts of the head and the tail, female); NHMUK PV P 2099a (specimen lacking the rostrum and parts of the disc, female); MCZ-317 (partly preserved specimen lacking the left pectoral fin, parts of the pelvic region and the tail, presumably female); MGL 9-312 (specimen with the entire body outline preserved but lacking skeletal structures of the head and disc, male); MNB 12251 (specimen with only the head and parts of the disc preserved, unknown sex); TM 14838 (almost complete specimen lacking parts of the tail, female);

*Diagnosis.* Guitarfish-like batomorph; unique in the combination of the following characters: elongated, spatula-shaped rostral appendix present; antorbital cartilages present but reaching less than half of the distance between the nasal capsules and the propterygium; 38–40 pectoral radials (8–9 propterygial, 8–9 mesopterygial, *c*. 21 metapterygial); one pectoral radial articulating directly with the scapulocoracoid between the meso- and metapterygium; the pectoral radials segmented in up to five segments; the pelvic girdle strongly curved; at least 19 basipterygial radials (including one compound radial); the claspers are long, fairly robust, with a knob-like terminal portion; minute fin spines associated with the dorsal fins.

*Type locality.* Upper Kimmeridgian; Cerin, Ain, France.

### Description

*General body form.* Medium-sized batomorph characterized by a guitarfish-like body shape, reaching a total length of *c*. 60 cm. The pectoral fins are fused to the head, and the elongated snout creates a drop-shaped disc. The disc reaches on average a width of 39.3% of the total length (range, 35.4–41.9% TL) and a length of 46.2% TL (range, 40.6–50.6% TL).

*Neurocranium.* The neurocranium reaches its maximum width at the level of the nasal capsules, narrows at the orbits, and widens again at the postorbital processes. Towards the synarcual, in the otic region, the neurocranium becomes slightly narrower again. The rostral cartilage is relatively long and accounts for *c*. 14.8% TL (range, 13.4–16.3% TL). The rostrum is longer than the remaining part of the neurocranium and narrows at midlength. In dorsal view a precerebral fossa is present. A rostral appendix is attached to the anteriormost portion of the rostral cartilage, giving the snout a spatula-like shape. The nasal capsules are oval, smooth and lack horn-like processes. They are slightly less broad than the jaw cartilages. No preorbital processes are discernible. Attached to the posterior surface of the nasal capsules are the antorbital cartilages, which are narrow and directed lateroposteriorly. They are short and extend less than halfway between the nasal capsules and the propterygium. The postorbital processes are short and not very distinct.

*Jaws & branchial skeleton.* A distinct symphysis is visible in both the palatoquadrate and the Meckel’s cartilage where the jaw rami meet. The jaws are curved and wider distally than mesially. The dorsal flange of the Meckel’s cartilage is broad and anterolaterally oriented. The ceratobranchials are long and plate-like. A narrow and long basihyal is present.

*Axial skeleton & unpaired fins.* The synarcual contains large and fully developed vertebral centra along most of its length. The synarcual lip fits well into the notch in the basicranium. The vertebral centra are cyclospondylic and similar in size over most of the specimens, gradually becoming smaller only in the second half of the tail. The supraneural spines are well-developed and plate-like. Several pairs of ribs are present. Two small fin spines are associated with the dorsal fins.

*Pectoral girdle & fins.* The propterygium is crescent-shaped and attached to it are 8–9 radials. Another 8–9 radials are connected to the oval mesopterygium, which is tangential to the propterygium along its entire length. A gap is present between the mesopterygium and metapterygium, enabling one radial to connect directly with the scapulocoracoid. The metapterygium is long, narrow and curved and has *c*. 21 radials attached to it. The radials are divided into five segments.

*Pelvic girdle, fins & claspers.* The puboischiadic bar is curved and has distinct lateral prepelvic processes. The anterior compound radial is directed posterolaterally and followed by *c*. 18 radials. The tail length (distance from pelvic girdle to tip of caudal fin) averages 60.3% TL and appears to increase during ontogeny, with juvenile specimens of *c*. 30 cm TL having a tail length of *c*. 57.6% TL (range, 57.3–57.9% TL) and adult specimens (58.2 cm TL), of *c*. 65.6% TL.

The claspers (i.e. mixopterygia) are relatively long, reaching approximately to the origin of the first dorsal fin or slightly beyond. They measure a length of *c*. 21 vertebral centra (MGL 9-312) and are fairly robust. The terminal ends of the claspers are knob-like. In the juvenile male (MDC 20015298) they are not yet very pronounced and can be recognized by the fact that they are longer than the last pelvic radial, protrude slightly beyond the pelvic fin, and have a slight thickening at the tip.

*Teeth & denticles.* Teeth from holomorphic *Spathobatis bugesiacus* specimens (NHMUK PV P 2099, MDC 20015308) were described by Cavin *et al*. (1995): the crown of the teeth is about as long as wide. The teeth have a well-developed transversal crest that separates the labial from the lingual face of the crown. A distinct median cusp is present. The labial apron is not very large but distinct and peg-like. The lingual uvula, however, is well-developed and massive. The holaulacorhize root is thick, has broad lobes, and is lingually displaced.

The dermal denticles have a flat and star-shaped base and either a knob-like or an elongated, arrow-shaped crown (Thies & Leidner 2011).

### Comparison

Elongated, spatula-shaped rostral appendix present (unlike *B. sismondae* and *K. etchesi*, but similar to *A. bavarica* and *As. platypterus*). Antorbital cartilages are present (unlike *B. sismondae*) but reach less than half of the distance between the nasal capsules and the propterygium (unlike *As. platypterus* but like *A. bavarica* and *K. etchesi*). One pectoral radial is articulating directly with the scapulocoracoid between the meso- and metapterygium (unlike *A. bavarica, As. platypterus, B. sismondae* and *K. etchesi*). The pectoral radials are segmented in up to five segments (unlike *As. platypterus*, but like *A. bavarica*). The pelvic girdle is strongly curved (unlike *As. platypterus* and *B. sismondae*, but like *A. bavarica*). Fin spines are associated with the dorsal fins (unlike *A. bavarica* and *K. etchesi*, but like *As. platypterus* and *B. sismondae*); the spines are minute (unlike *B. sismondae*, but like *As. platypterus*).

## Traditional Morphometrics

### General

**Fig. 9:**
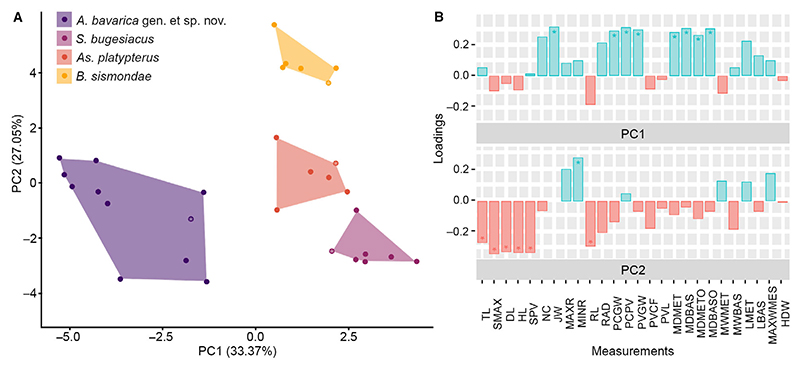
Traditional morphometrics: results of the principal component analysis (PCA) performed on the entire dataset, with each measurement adjusted to percentage of the disc width (DW) of each individual. A, morphospace plotted on PC1 (33.37%) and PC2 (27.05%). Asterisks indicate the holotype of the respective species. B, loading values showing the variables associated with the first two PC axes. Asterisks indicate the variables that explain the most variation for the respective PC axes. *Abbreviations*: DL, disc length; HDW, half disc width; HL, head length; JW, jaw width; LBAS, length of basipterygia; LMET, length of metapterygia; MAXR, maximum rostrum width; MAXWMES, maximum width of mesopterygia; MDBAS, inner maximum distance between basipterygia; MDBASO, outer maximum distance between basipterygia; MDMET, inner maximum distance between metapterygia; MDMETO, outer maximum distance between metapterygia; MINR, minimum rostrum width; MWBAS, maximum width of basipterygia; MWMET, maximum width of metapterygia; NC, nasal capsules maximum width; PCGW, pectoral girdle width; PCPV, pectoral girdle to pelvic girdle; PVCF, pelvic girdle to caudal fin tip; PVGW, pelvic girdle width; PVL, pelvic fin length; RAD, span between anteriormost fin radials; RL, rostrum length; SMAX, distance from the tip of the snout to the point of maximum disc width; SPV, snout to pelvic girdle; TL, total length.

A total of 27 measurements were taken along the entire body of each of the 30 best preserved specimens (Table S1; Fig. 1) representing each of the identified species except *Kimmerobatis etchesi* (Material and Method, above), which were then adjusted to percentage of the disc width. With this dataset we performed a PCA considering all measurements, which resulted in 26 axes (Table S4), with the first four each explaining more than 5% of the variation and together accounting for 77.59% of the total variability (Table S5). The most important variables for PC1 and PC2 are described in detail later in this section. The occupied morphospace plotted on the first two axes and the associated variables are shown in Figure 9. The four species groups are well separated along PC1 (33.37%) and PC2 (27.05%). The morphospaces occupied by *Asterodermus platypterus* and *S. bugesiacus* are situated relatively close to each other, while the morphospaces of *A. bavarica* and *B. sismondae* are clearly separated from all others. It appears, however, that two intraspecies-level groups are present within *A. bavarica*, which occupies a rather wide morphospace (Fig. 9A). To verify the initial grouping we performed an LDA, which aims to find the information that separates the groups the best, and assigned the two putative *A. bavarica* clusters to different hypothetical groups. The results of the LOOCV show that the specimens are associated with both groups, but never with any of the other species, confirming their assignment to a single *A. bavarica* group (Fig. S3, Table S6).

The results of the Shapiro–Wilk normality test showed that *c*. 76.92% of all measurements are normally distributed (i.e. 20 out of 26 measurements; Table S7). The non-normally distributed measurements were further analysed using the Kruskal–Wallis rank sum test, which indicates significant differences between the taxa for all but one measurement (i.e. maximum width of mesopterygium; Table S8). Pairwise Wilcoxon tests between the taxa also suggest significant differences (Table S9). ANOVA tests on each normally distributed measurement showed significant differences in all but three measurements (i.e. maximum width of basipterygia, length of basipterygia, and half disc width; Table S10), which was also largely confirmed by pairwise comparisons (Table S11).

Additionally, a subset including only normally distributed values was generated. The PCA using this subset resulted in 20 axes (Table S12), with the first five accounting for 83.42% of the total variation (Table S13). The other 15 axes each account for <5% of the total variation. The morphospace occupation by the different groups plotted on the first two axes is markedly similar to that obtained using both normally and non-normally distributed measurements (Fig. S4).

*Strongest vectors for PC1.* The following measurements were identified as explaining the greatest variation along PC1 (33.37% of the total variation) and are compared between species below: jaw width (JW), distance from pectoral girdle to pelvic girdle (PCPV), maximum outer distance between basipterygia (MDBASO), maximum inner distance between basipterygia (MDBAS), pelvic girdle width (PVGW), pectoral girdle width (PCGW), maximum outer distance between metapterygia (MDMETO) and maximum inner distance between metapterygia (MDMET).

**Fig. 10:**
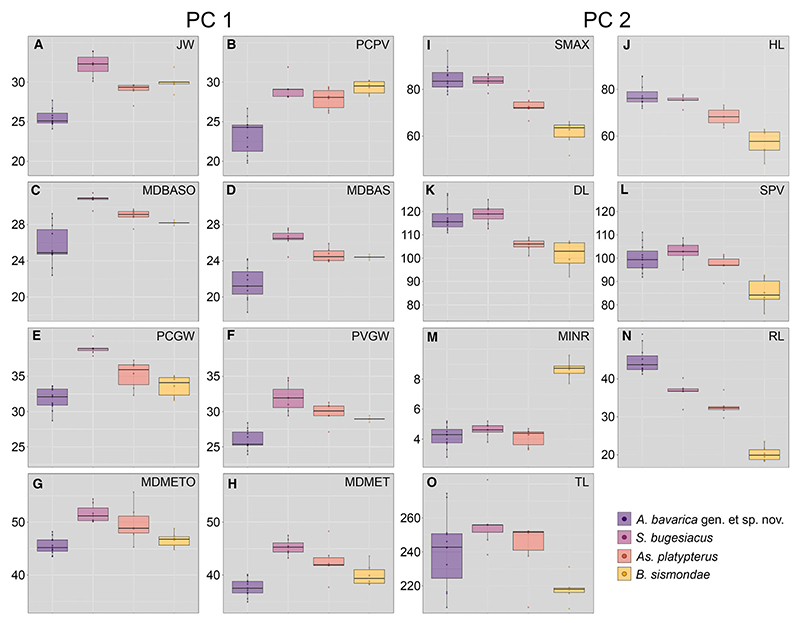
Traditional morphometrics: boxplots of the variables explaining the most variation of principal component (PC)1 and PC2, respectively. Measurements are adjusted as percentage of the disc width (DW). A, jaw width. B, pectoral girdle to pelvic girdle. C, outer maximum distance between basipterygia. D, inner maximum distance between basipterygia. E, pectoral girdle width. F, pelvic girdle width. G, outer maximum distance between metapterygia. H, inner maximum distance between metapterygia. I, tip of snout to the point of maximum disc width. J, head length. K, disc length. L, snout to pelvic girdle. M, minimum rostrum width. N, rostrum length. O, total length.

JW ranges from 24.1% to 27.7% disc width (DW) in *A. bavarica*, from 27.0% to 29.1% DW in *As. platypterus*, from 28.4% to 31.9% DW in *B. sismondae*, and from 30.1% to 33.9% DW in *S. bugesiacus*. The pairwise comparison shows that all taxa are significantly different in this relative measurement, except for *B. sismondae* to *As. platypterus* and to *S. bugesiacus* (Table S11, Fig. 10A).

PCPV spans from 19.8% to 26.7% DW in *A. bavarica*, from 26.1% to 29.4% DW in *As. platypterus*, from 28.2% to 30.2% DW in *B. sismondae*, and from 28.1% to 31.9% DW in *S. bugesiacus*. The pairwise Wilcoxon test indicates that *A. bavarica* can be significantly separated from all other taxa, however, *As. platypterus, B. sismondae* and *S. bugesiacus* are not significantly different from each other in this relative measurement (Table S9, Fig. 10B).

MDBASO ranges from 22.4% to 29.2% DW in *A. bavarica*, from 27.5% to 29.7% DW in *As. platypterus*, from 27.9% to 28.5% DW in *B. sismondae*, and from 29.5% to 31.5% DW in *S. bugesiacus*. According to the pairwise Wilcoxon test, *S. bugesiacus* is the most distinct taxon of all as to this relative measurement, being significantly different from all others. Only the statistical differentiation of *B. sismondae* and *A. bavarica* as well as *B. sismondae* and *As. platypterus* is not possible with the present dataset (Table S9, Fig. 10C).

The intraspecies range of MDBAS is similar to that of MDBASO: it ranges from 18.3% to 24.2% DW in *A. bavarica*, from 23.9% to 25.9% DW in *As. platypterus*, from 24.1% to 24.7% DW in *B. sismondae*, and from 24.4% to 27.6% DW in *S. bugesiacus*. However, unlike the results for MDBASO, pairwise comparison shows that *A. bavarica* is significantly different from all other taxa, while *As. platypterus, B. sismondae* and *S. bugesiacus* cannot be statistically significantly differentiated based on this relative measurement (Table S11, Fig. 10D).

PCGW ranges from 28.7% to 33.6% DW in *A. bavarica*, from 32.3% to 37.3% DW in *As. platypterus*, from 31.6% to 35.1% DW in *B. sismondae*, and from 37.9% to 40.7% DW in *S. bugesiacus*. The pairwise comparison significantly separates all taxa from each other except *B. sismondae* from *A. bavarica* and *As. platypterus* (Table S11; Fig. 10E).

PVGW spans from 23.9% to 28.4% DW in *A. bavarica*, from 27.1% to 31.3% DW in *As. platypterus*, from 28.5% to 29.4% DW in *B. sismondae*, and from 29.4% to 34.8% DW in *S. bugesiacus*. Pairwise comparison indicates significant differences of *A. bavarica* to all other taxa as well as between *B. sismondae* and *S. bugesiacus* (Table S11; Fig. 10F).

MDMETO ranges from 43.6% to 48.2% DW in *A. bavarica*, from 45.3% to 55.7% DW in *As. platypterus*, from 44.8% to 48.8% DW in *B. sismondae*, and from 50.1% to 54.4% DW in *S. bugesiacus*. Similarly, MDMET spans from 34.9% to 40.1% DW in *A. bavarica*, from 37.7% to 48.3% DW in *As. platypterus*, from 38.2% to 43.6% DW in *B. sismondae*, and from 43.2% to 47.5% DW in *S. bugesiacus*. Both pairwise comparisons show significant differences between *A. bavarica* and both *As. platypterus* and *S. bugesiacus*, as well as between *B. sismondae* and *S. bugesiacus* (Table S11; Fig. 10G, H).

### Strongest vectors for PC2

For PC2 (27.05% of the total variation), the following measurements explained the most variation and are thus compared between species below: distance from the tip of the snout to the point of maximum disc width (SMAX), head length (HL), disc length (DL), distance from the tip of the snout to the pelvic girdle (SPV), minimum rostrum width (MINR), rostrum length (RL) and total length (TL).

SMAX ranges from 77.8% to 96.6% DW in *A. bavarica*, from 66.5% to 79.3% DW in *As. platypterus*, from 51.7% to 66.2% DW in *B. sismondae*, and from 78.3% to 86.8% DW in *S. bugesiacus*. Pairwise comparison indicates significant differences between the taxa, except between *A. bavarica* and *S. bugesiacus*, and between *As. platypterus* and *S. bugesiacus* (Table S11; Fig. 10I).

HL ranges from 71.9% to 85.6% DW in *A. bavarica*, from 63.5% to 73.2% DW in *As. platypterus*, from 48.2% to 62.9% DW in *B. sismondae*, and from 71.2% to 77.7% DW in *S. bugesiacus*. A pairwise Wilcoxon test shows that all taxa are significantly different from each other, with the exception of *A. bavarica* to *S. bugesiacus* (Table S9; Fig. 10J).

DL spans from 110.9% to 127.6% DW in *A. bavarica*, from 101.0% to 108.9% DW in *As. platypterus*, from 92.0% to 107.3% DW in *B. sismondae*, and from 112.6% to 125.1% DW in *S. bugesiacus*. Pairwise comparison indicates significant differences between the taxa, except between *A. bavarica* and *S. bugesiacus*, and between *As. platypterus* and *B. sismondae* (Table S11; Fig. 10K).

SPV spans from 91.9% to 111.1% DW in *A. bavarica*, from 89.2% to 101.5% DW in *As. platypterus*, from 76.2% to 92.7% DW in *B. sismondae*, and from 95.0% to 108.7% DW in *S. bugesiacus*. Pairwise comparison shows that *B. sismondae* is significantly different from all other taxa (Table S11; Fig. 10L).

MINR reaches similar values in all taxa except *B. sismondae*. It ranges from 2.8% to 5.2% DW in *A. bavarica*, from 3.3% to 4.7% DW in *As. platypterus*, from 7.7% to 9.6% DW in *B. sismondae*, and from 3.8% to 5.2% DW in *S. bugesiacus*. Accordingly, the pairwise Wilcoxon test shows significant differences between *B. sismondae* and all other taxa (Table S9; Fig. 10M).

RL ranges from 41.2% to 51.7% DW in *A. bavarica*, from 29.7% to 37.1% DW in *As. platypterus*, from 18.3% to 23.5% DW in *B. sismondae*, and from 31.9% to 40.2% DW in *S. bugesiacus*. Pairwise comparison indicates significant differences between the taxa, except between *A. bavarica* and *S. bugesiacus*, and between *As. Platypterus* and *S. bugesiacus* (Table S11; Fig. 10N).

TL ranges from 207.2% to 274.4% DW in *A. bavarica*, from 207.3% to 251.1% DW in *As. platypterus*, from 206.4% to 231.1% DW in *B. sismondae*, and from 238.3% to 282.4% DW in *S. bugesiacus*. pairwise comparison shows that *B. sismondae* is significantly different from all other taxa, while *A. bavarica, As. platypterus* and *S. bugesiacus* cannot be statistically significantly separated by this relative measurement (Table S11; Fig. 10O).

## Geometric Morphometrics

**Fig. 11:**
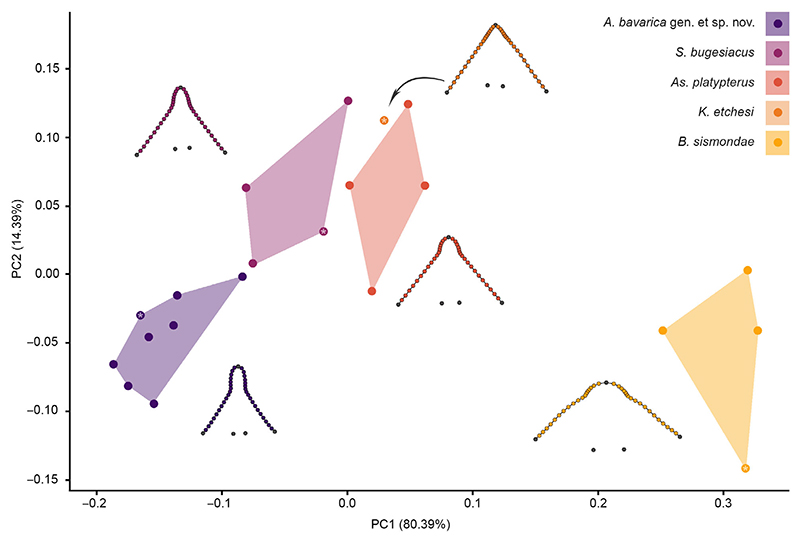
Geometric morphometrics: results of the principal component analysis (PCA). Morphospace plotted on PC1 (80.39%) and PC2 (14.39%). Asterisks indicate the holotype of the respective species. Mean shapes are shown next to each group. Dark grey dots of the mean shapes indicate true landmarks, coloured dots indicate semilandmarks.

In total, the snout region of 21 specimens (Table S1) was analysed using five landmarks and 36 semilandmarks. All identified species are represented in this analysis. *Kimmerobatis etchesi* is included in the morphospaces only for graphical representation of its placement, but not in the statistical analyses (Material and Method, above). A PCA was performed on the 2D landmark dataset, resulting in 20 axes, with the first two accounting for 94.79% of the total shape variation (Table S14). The remaining 18 axes each account for <5% of the total variation. The positive area of PC1 (80.39%) is occupied by specimens with a broad head and a rounded snout, while negative values indicate specimens with a rather narrow head and an elongated rostrum. In the positive area of PC2 (14.39%) there are specimens with a pointed or only slightly elongated rostrum (Fig. 11).

In contrast to the morphospace occupation obtained with the traditional morphometrics dataset (Fig. 9A), *A. bavarica* is not divided into two putative groups. Nevertheless, we classified the *A. bavarica* specimens present in both datasets into the same hypothetical groups and performed an LDA to analyse possible morphotypes, resulting in both groups clustering together (Fig. S5; Table S15).

**Fig. 12:**
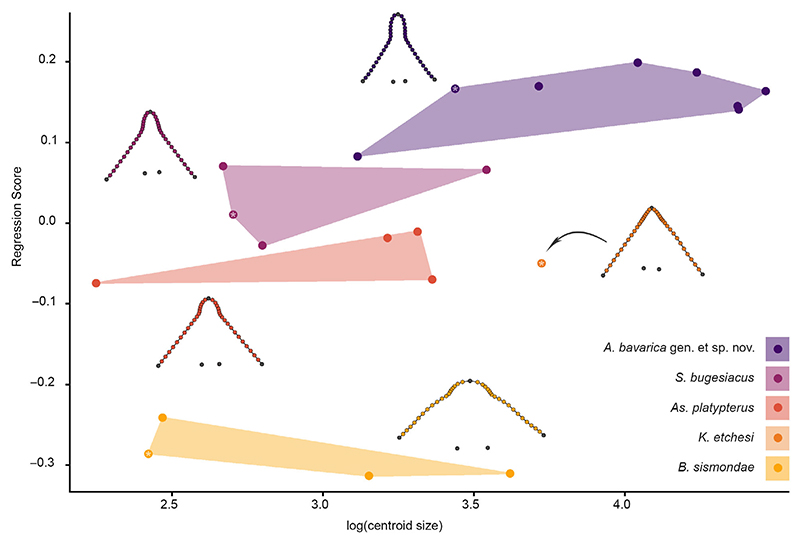
Log(centroid size) versus the multivariate regression of the Procrustes coordinates (regression score) of shape for each group. Asterisks indicate the holotype of the respective species. Mean shapes are shown next to each group. Dark grey dots of the mean shapes indicate true landmarks, coloured dots indicate semilandmarks.

As shown using a Procrustes ANOVA, the size of the specimens has an influence on the shape of the rostrum (*R*^2^ = 0.26484, *F* = 6.8447, *Z* = 2.252, p = 0.008; Table S16). However, the linear regression of the Procrustes coordinates on the logarithm of the centroid size also shows that different rostral shapes assigned to different species are separated from each other, even if the specimens are similar in size (Fig. 12). Another Procrustes ANOVA indicates significant differences in shape between the taxa (*R*^2^ = 0.87995, *F* = 29.319, *Z* = 6.4229, p = 0.001; Table S17). The pairwise comparison shows that these differences exist especially between *B. sismondae* and all other taxa, but also between *A. bavarica* and *As. platypterus* (Table S18). ANOSIM confirms the significant differences between all tested taxa (*R* = 0.9052, p = 0.0001), especially between *As. platypterus* and *A. bavarica*, between *B. sismondae* and *A. bavarica*, as well as between *S. bugesiacus* and *A. bavarica* (Table S19). Finally, we performed a PERMANOVA (*F* = 47.68, p = 0.0001) to test for differences between the group centroids and obtained the same results as in the ANOSIM (Table S20).

## Discussion

We applied both qualitative and quantitative approaches to examine Late Jurassic batomorphs from various European localities. The different approaches yielded similar and complementary results. In total, we were able to identify all four holomorphic batomorph taxa known from Europe (i.e. *Asterodermus platypterus, Belemnobatis sismondae, Kimmerobatis etchesi, Spathobatis bugesiacus*), characterized by different snout and body shapes. Additionally, the presence of a previously undescribed taxon, *Aellopobatis bavarica*, was shown in both qualitative and quantitative analyses.

Previously, three holomorphic Late Jurassic European batomorph genera were unequivocally considered valid: *Belemnobatis, Kimmerobatis* and *Spathobatis*. Here, we show that at least two additional holomorphic taxa occurred during this period, namely *Asterodermus*, a genus formerly considered doubtful, the validity of which we confirm with this study, and *Aellopobatis*, which previously was regarded to be a large morphotype of *Spathobatis*.

Batomorphs from the Solnhofen Archipelago have previously been referred to as both *As. platypterus* (e.g. Kriwet & Klug 2004; Thies & Leidner 2011) and *S. bugesiacus* (e.g. Frickhinger 1999; Kriwet & Klug 2015), but never with solid morphological justification. In this study we found no evidence for the presence of *S. bugesiacus* in the Solnhofen Archipelago. However, our results indeed show the presence of *As. platypterus* and a second, hitherto unknown batomorph species, which is characterized by its extremely elongated snout, its large size and the absence of fin spines. We have named this species, so far only known from the lower Tithonian of the Solnhofen Archipelago, *Aellopobatis bavarica*. In addition, the analysis based on body proportions (i.e. traditional morphometrics) indicates the presence of two morphotypes of *A. bavarica* (Fig. 9). However, no qualitative characters were found to distinguish between them, and also an LDA did not separate the two groups (Fig. S3; Table S6). In modern skates, sexual dimorphism of the pectoral fin shape is known to occur in adult male and female specimens (Martinez *et al*. 2019). However, given that both potential morphotypes of *A. bavarica* contain both sexes as well as specimens of different sizes, a separation caused by sexual or ontogenetic dimorphism can be excluded. Some of the same specimens included in this analysis were also included in the geometric morphometric analysis on the snout shape, but no such separation could be detected there. In extant elasmobranchs, it is common to identify new species mainly on the basis of morphometric data such as body proportions, sometimes in combination with other approaches such as genetic or life history studies (e.g. Iglésias *et al*. 2010). Similarly, extant cryptic species have been identified using molecular data (e.g. Naylor *et al*. 2012) and morphometric analyses (e.g. Aroca *et al*. 2022). However, it is not possible to genetically confirm the presence of cryptic Late Jurassic species, thus it is necessary to rely on morphological and meristic data to distinguish species. An important aspect of species differentiation that is standard in palaeontology but has little appeal to neontologists is the study of tooth morphology (Guinot *et al*. 2018); detailed knowledge of tooth morphology has the potential to distinguish cryptic species from each other. Therefore, future studies that investigate the teeth of the different holomorphic batomorphs in detail could clarify whether the morphospace occupation by *A. bavarica* is due to the presence of two cryptic species or whether it merely represents intraspecies variation.

Given the lack of the complete cranial region of the holotype, the validity of the monotypic batomorph genus *Asterodermus* was debated in the past (Underwood & Rees 2002). Agassiz (1836) introduced the name *Asterodermus platypterus* for this headless specimen and used the star-shaped outline of the placoid scales to characterize the taxon. However, the star-shaped bases of the roots of the placoid scales are not useful morphological features, given that the placoid scale base is also star-shaped in other batomorphs, such as *Spathobatis bugesiacus* (Leidner & Thies 1999). Leidner & Thies (1999) assumed that all Upper Jurassic batomorphs from southern Germany belong to this genus based on the morphology of the placoid scales, an opinion followed by Kriwet & Klug (2004). However, Kriwet & Klug (2015) reconsidered their earlier interpretation and posited the occurrence of both *As. platypterus* and *S. bugesiacus* in the Solnhofen Archipelago. Until now, dental morphology has been the primary means of distinguishing between Late Jurassic batomorphs, making the unequivocal identification of *As. platypterus* impossible because these characters are absent in the holotype. Underwood and Rees (2002) therefore suggested that *As. platypterus* should be considered a *nomen dubium* until diagnostic skeletal characters are described. Here, we show that the use of different methods for species discrimination can be highly useful when one approach alone (e.g. the qualitative study of morphological characters) is not sufficient for certain reasons. In our study, we found several batomorph specimens with skeletal features matching those seen in the holotype specimen of *As. platypterus* (i.e. straight form of the pelvic girdle, articulation of all radials with the basal cartilages, four radial segments, small fin spines). Additionally, body proportions (i.e. traditional morphometrics) also supported the assignment of these specimens to *As. platypterus*, and furthermore they differed significantly from other taxa in their snout shape (i.e. geometric morphometrics). This enabled us to allocate these specimens to *As. platypterus*, which helped to identify additional features in the head region (i.e. knob-like rostral appendix forming a slightly elongated rostrum, antorbital cartilages that extend up to halfway from the nasal capsules to the propterygium, teeth without distinct labial apron) to distinguish *As. platypterus* from other spathobatids and ultimately confirm the validity of this genus and species.

We exclude the possibility that the different taxa represent ontogenetic variations of the same species for two main reasons: First, we were able to study different sizes and thus different ontogenetic stages of all here presented taxa, with juvenile specimens having the same interspecies differences to the other taxa as adults. In *Spathobatis bugesiacus* we observed a juvenile male specimen with small, still developing claspers and a presumably adult male specimen with fully developed claspers; the putative adult male had a total length of just under 60 cm and was thus less than half the size of an adult male of *Aellopobatis bavarica* (*c*. 170–175 cm). Extant elasmobranchs typically become sexually mature when they have already reached more than 50% of their total length (e.g. Martins *et al*. 2018); the well-developed and long claspers of the 60-cm-long male *Spathobatis bugesiacus* indicate that it is an adult individual that has either already reached its total length or would not grow larger than 90 cm maximum, and thus would not reach a size of *c*. 170–175 cm. Second, it is improbable that *A. bavarica* represents an adult form of *As. platypterus* or *S. bugesiacus* because, unlike the latter two taxa, it lacks fin spines. In extant cartilaginous fishes, the loss of fin spines during ontogeny is not known (e.g. Maisey 1979), and so far there is no evidence of such a dramatic ontogenetic morphological change in extinct taxa.

All of the known holomorphic specimens of Late Jurassic batomorphs, other than the South American specimen (Cione 1999), are from a small palaeogeographical area covering what is now central and northern Europe. This was an area of epicontinental seas spanning areas with a largely Tethyan to largely Boreal oceanic influence. In addition, they all originate from a narrow stratigraphic range, spanning the upper part of the Kimmeridgian to the lower part of the Tithonian. These holomorphic Late Jurassic batomorphs are each known only from a single site, or in the case of the Solnhofen area, single region. Although each of these sites is unique in terms of palaeoenvironment, associated taxa, including neoselachian and hybodont sharks, are commonly found in multiple sites across Europe (e.g. Stumpf *et al*. 2022; Villalobos-Segura *et al*. 2023). It therefore appears that the Late Jurassic spathobatids had greater palaeoenvironmental specificity than other chondrichthyans of the time. Palaeoecological specificity was well developed within batomorphs and neoselachian sharks in the Middle Jurassic (Underwood 2004; Underwood & Ward 2004), and it is probable that this was typical of Jurassic chondrichthyan faunas. With individual batomorph taxa occupying only narrowly defined palaeoecological niches, it is probable that the global diversity of Middle and Late Jurassic batomorphs was very high, despite all known taxa possessing a very conservative body form. Given that completely preserved fossil Chondrichthyes are relatively rare compared with the abundance of fossil teeth, the study of tooth morphology provides an opportunity to further explore the potentially high diversity of Jurassic batomorphs. Findings of such isolated teeth, however, indicate a widespread occurrence of the genera *Belemnobatis* and *Spathobatis*, not only of the species *B. sismondae* and *S. bugesiacus* but also of other species not yet known as holomorphic specimens. However, given the new insights from the present study, the resulting increase in diversity of holomorphic species, and the apparent local occurrence of taxa, it is now necessary to re-evaluate isolated teeth. If necessary, genus or species affiliation must be revised to obtain a better understanding of past diversity and palaeogeographic patterns of Late Jurassic batomorphs.

Teeth from holomorphic specimens of *Belemnobatis sismondae* and *Spathobatis bugesiacus* were described by Saint-Seine (1949) and Cavin *et al*. (1995), respectively. Thies & Leidner (2011) further studied the tooth morphology of ‘*Spathobatis bugesiacus*’ (NHMUK PV P 10934), however, according to our current results, the specimen clearly belongs to *As. platypterus*; thus, we do possess information on the tooth morphology of *As. platypterus*, which was previously considered a *nomen dubium* given that the head region and thus the teeth are missing in the holotype (Underwood & Rees 2002). The newly described species *Aellopobatis bavarica* was formerly thought to be a large morphotype of *S. bugesiacus*, in contrast to our present results, which confirm its validity as a separate species. Tooth morphologies of holomorphic specimens are known from Thies & Leidner (2011), who described the teeth of two specimens of ‘*Asterodermus* sp.’ (SNSB-BSPG 1964 XXIII 577 and SNSB-BSPG AS I 1377), which we assign to *A. bavarica* based on our analyses. The comparison of the teeth of these specimens shows that species discrimination based on tooth morphology appears to be possible. The teeth of *S. bugesiacus* differ markedly from those of the other taxa: *Spathobatis bugesiacus* teeth have a welldeveloped median cusp, the crown is roughly as long as broad, the labial apron is peg-like and distinct, and the lingual uvula is massive. Teeth of *B. sismondae* have a weakly developed median cusp, a small and detached apron, and a narrow uvula. Teeth of *As. platypterus*, however, lack a distinct median cusp, are mesiodistally expanded, possess a small apron only vaguely detached from the crown, and a bulbous uvula that is never as massive as in *S. bugesiacus*. The teeth of *A. bavarica* resemble those of *As. platypterus* in that they have a rudimentary median cusp, a mesiodistally expanded tooth crown, and a narrow but bulbous uvula. However, the apron is not separated from the crown and can only be suggested by the rounded contour of the labial tooth face.

Although the dental morphology of individual specimens of each holomorphic taxon except *Kimmerobatis etchesi* is well known, a larger sample size within all species is required to fully understand intra- and interspecies dental diversity; elasmobranchs are known to show various types of heterodonty (i.e. change in tooth shape in the jaw) between sexes of the same species, or during ontogeny (e.g. Compagno 1970; Berio *et al*. 2020; Türtscher *et al*. 2021, 2022), meaning that knowledge of the tooth morphology of individual specimens offers only a small glimpse at the big picture of dental variety of a species. Future quantitative studies on the teeth of more holomorphic spathobatids should be conducted to augment our understanding of the diversity and dental disparity of Late Jurassic batomorphs and to confidently assign isolated teeth to a specific genus or species.

The overall body form of all known Spathobatidae is very similar to that of the extant guitarfishes (Rhinobatidae, Glaucostegidae) and wedgefishes (Rhinidae) within the Rhinopristiformes. Indeed, the guitarfish morphology, consisting of a well-developed rostrum, somewhat elongate trunk, muscular caudal region and clearly differentiated caudal and dorsal fins, is present in all batomorph orders, as shown by *Zanobatus* in the Myliobatiformes, the Platyrhinidae in the Torpediniformes, and the Sclerorhynchoidea in the Rajiformes (Aschliman *et al*. 2012; Underwood & Claeson 2017; Villalobos-Segura *et al*. 2022). Likewise, the teeth of known spathobatid taxa can be differentiated from those of other batomorphs only by use of a combination of characters, and there are no morphological autapomorphies of the clade. It is therefore reasonable to presume that the guitarfish morphology and spathobatid-like tooth morphology are plesiomorphic in batomorphs. Although all holomorphic batomorphs described here form a monophyletic group, it should not be assumed that other mid-Mesozoic batomorphs with a similar body or tooth form would automatically also belong in the Spathobatidae unless they have been studied in detail, given that stem group batomorphs may well outwardly appear very similar. Studies on the so far oldest known holomorphic batomorph, an as yet undescribed specimen from the Early Jurassic of Germany (Maisey *et al*. 2020), will further enhance our knowledge of plesiomorphic characters of batomorphs and thus enable us to better understand the evolution of morphological traits in this successful group of cartilaginous fishes.

## Conclusion

Despite the excellent preservation of Late Jurassic holomorphic batomorphs, which enables detailed examination of body shapes and skeletal structures, they have remained largely understudied. Here, we provide the first comprehensive study of body shape variation in articulated spathobatids based on 52 specimens that originated from German, French and English Late Jurassic deposits. Our results show that it is possible to distinguish species based on the presence and shape of individual skeletal structures, body shape and body proportions using different methodological approaches (i.e. qualitative morphological descriptions and quantitative morphometric approaches). Instead of three, we can undoubtedly identify five species, and thus confirm a higher diversity of holomorphic batomorphs in the Late Jurassic. Furthermore, the different species seem to have been highly endemic, given that all species are so far known only from the respective region of their first description.

However, this interpretation is based on holomorphic specimens only. Up to now, our knowledge of the tooth morphologies of holomorphic batomorphs is very limited, but it seems that there are dental differences between the different species. More studies on the tooth morphologies of articulated batomorphs consequently are needed to establish solid morphological characters including intra- and interspecies variations for taxon discrimination, which ultimately will enable us to re-evaluate the taxonomic identity of isolated spathobatid teeth and, undoubtedly, to reconstruct their palaeogeographic occurrences. Given that only single species are known from each genus, the level of variation of dental morphology within genera is unclear. As a result of this, and the misidentification of figured teeth, genus-level identification of isolated teeth from the Jurassic and Early Cretaceous is likely to be problematic. The stratigraphical range of genera of the Spathobatidae is therefore essentially unknown other than in regard to the type species of these genera, the genus-level diversity of early batomorphs is likely to be severely underestimated, and hence isolated dental material should be reassessed in light of the genus-level diversity as recorded here.

## Supplementary Material

Additional Supporting Information can be found online (https://doi.org/10.1002/spp2.1552):

**Appendix S1.** Figs S1–S5 and R code.

**Appendix S2.** Tables S1–S20: list of examined specimens (S1), and results of traditional (S2–S13) and geometric (S14–S20) morphometric analyses.

Appendix S1

Appendix S2

## Figures and Tables

**Fig. 3:**
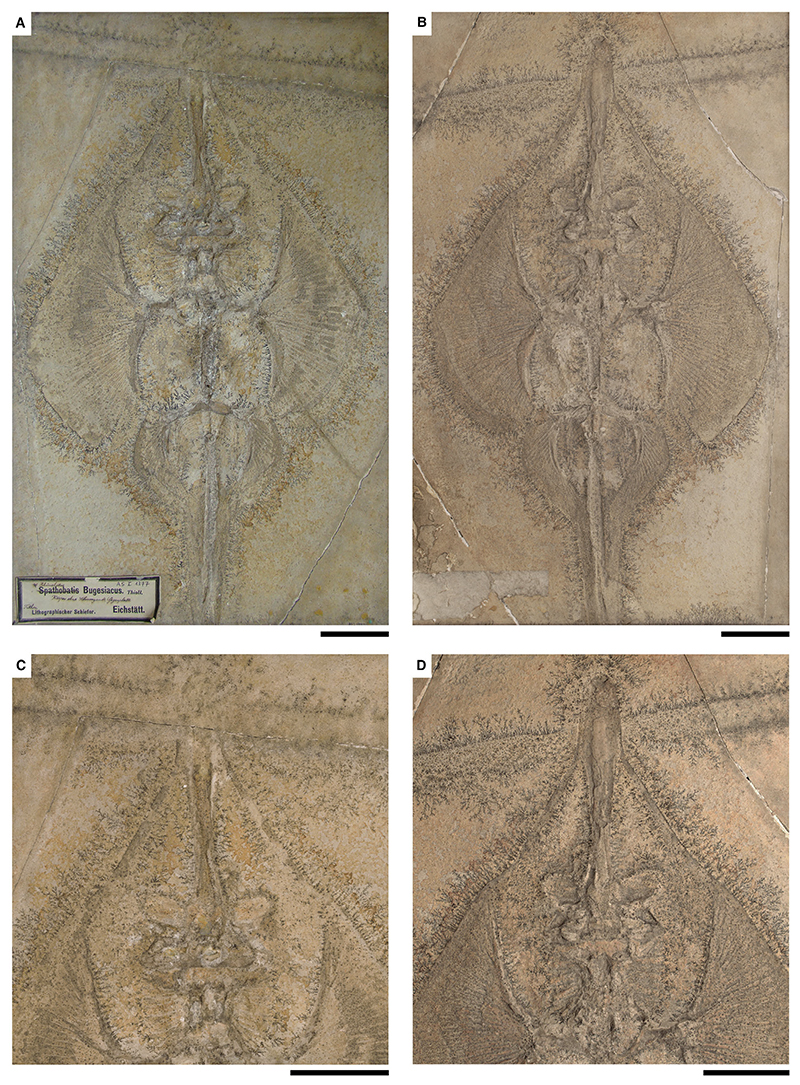
*Aellopobatis bavarica* gen. et sp. nov., holotype. A, SNSB-BSPG AS I 1377, complete specimen. B, SNSB-BSPG AS I 1378, complete specimen; C, SNSB-BSPG AS I 1377, close-up of the cranial region; D, SNSB-BSPG AS I 1378, close-up of the cranial region. Scale bars represent 5 cm.

**Fig. 4:**
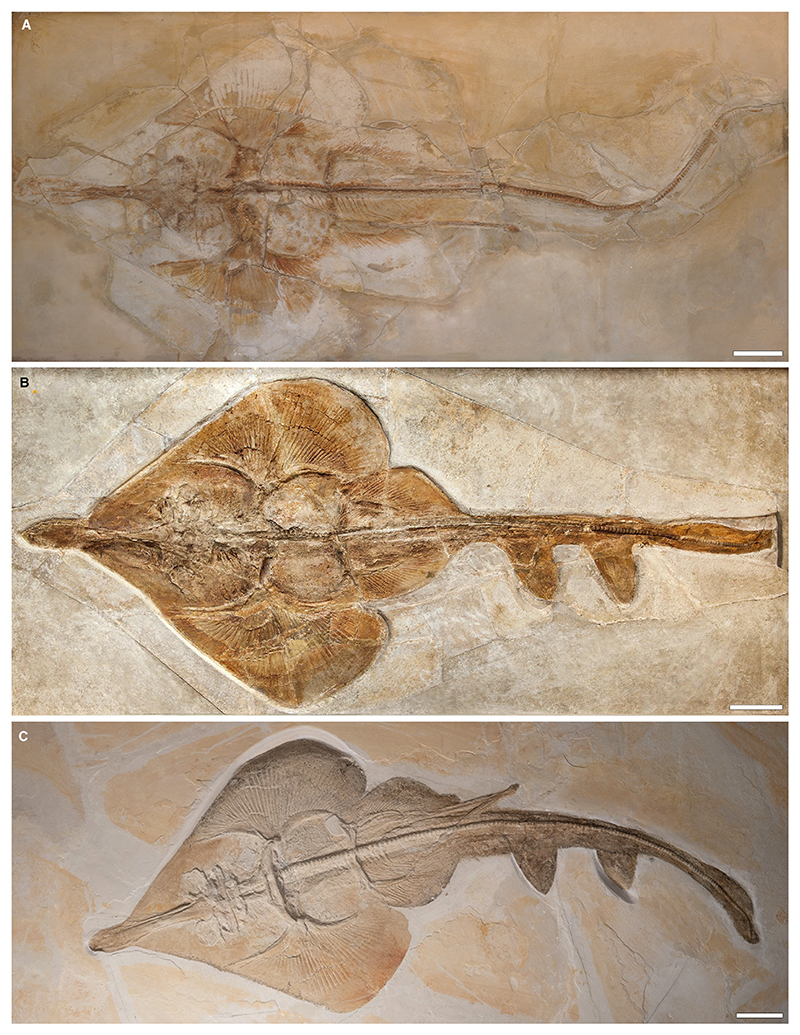
*Aellopobatis bavarica* gen. et sp. nov. A, CM 5396. B, NHMUK PV P 6010. C, LF 2323. Scale bars represent 10 cm.

**Fig. 5:**
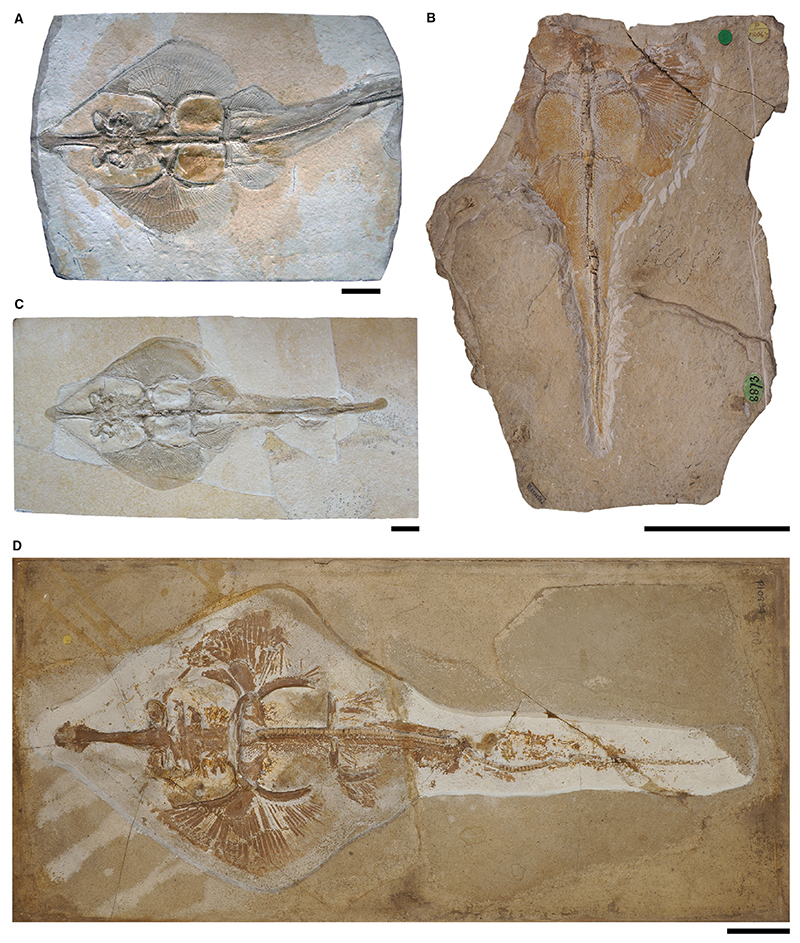
Asterodermus platypterus. A, JME SOS-3647. B, NHMUK PV P 12067, holotype. C, JME SOS-2212a. D, NHMUK PV P 10934. Scale bars represent 5 cm.

**Fig. 6:**
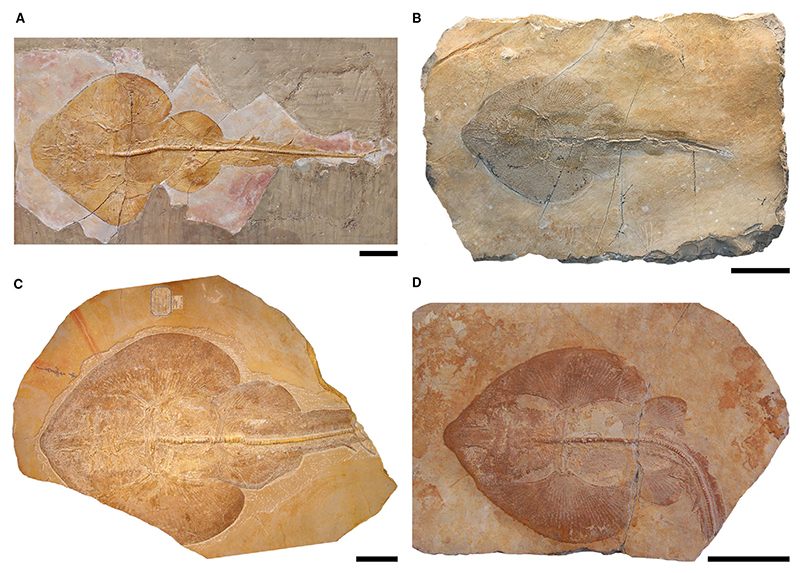
Belemnobatis sismondae. A, MGL 38-774. B, MDC 20015312. C, NRM P 1569. D, MDC 20015305, holotype (photo courtesy of Pierre Thomas, Musée des Confluences (Lyon, France), Planet-Terre (https://planet-terre.ens-lyon.fr/ressource/Img295-2009-12-07.xml)). Scale bars represent: 10 cm (A); 5 cm (B–D).

**Fig. 7:**
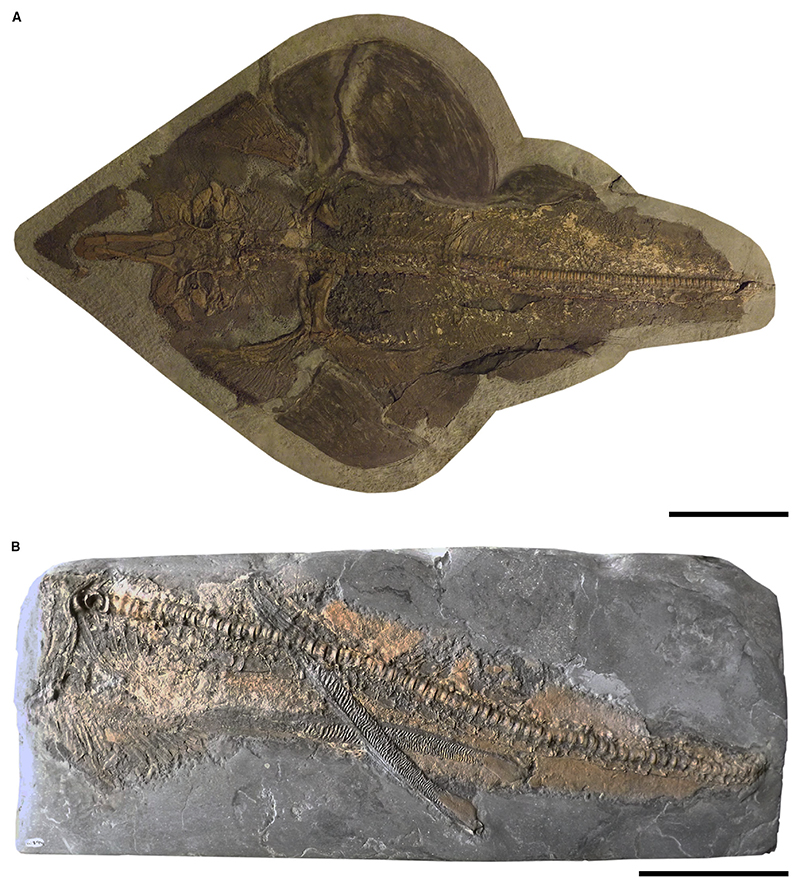
Kimmerobatis etchesi. A, MJML K874, holotype. B, MJML K1894, paratype. Scale bars represent 10 cm.

**Fig. 8:**
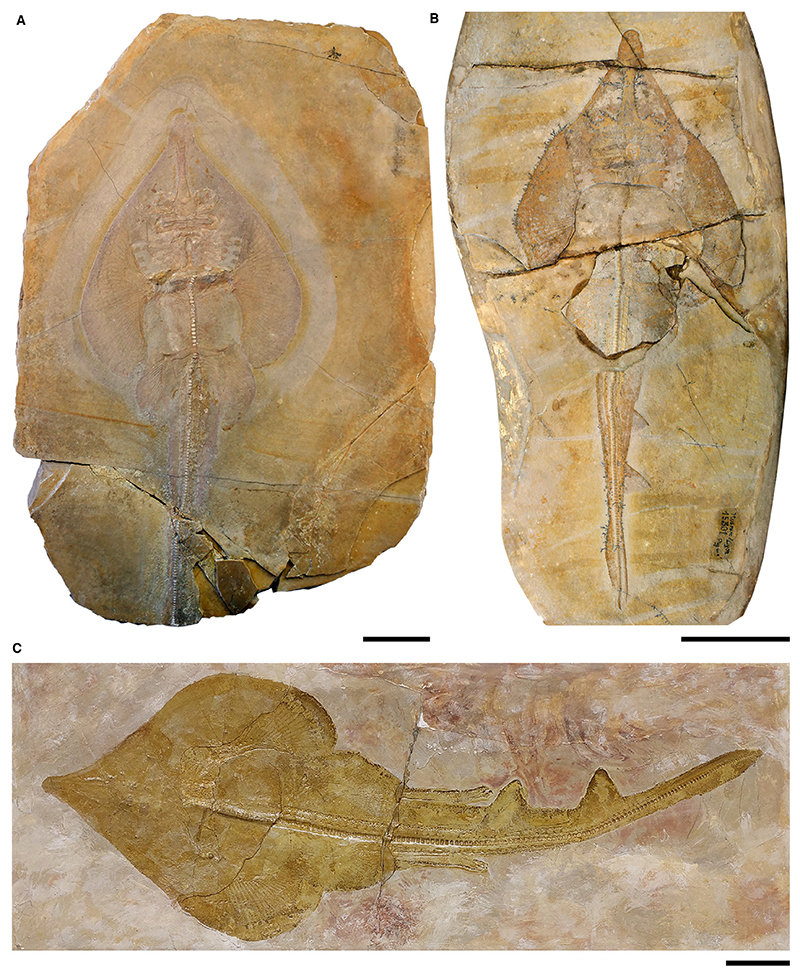
Spathobatis bugesiacus. A, MDC 20015307, holotype. B, MDC 20015301. C, MDC 20015312. Scale bars represent 5 cm.

## Data Availability

This published work and the nomenclatural acts it contains, have been registered in ZooBank: https://zoobank.org/References/82AA00A8-F3BD-4229-A344-BA7D5369901C *Editor*. Lionel Cavin
