# Supplementary material for: Rostral and body shape analyses reveal cryptic diversity of Late Jurassic batomorphs (Chondrichthyes, Elasmobranchii) from Europe
Source: Pap Palaeontol. Author manuscript; Available in PMC 2024 May 26. (PMC7615989; doi:10.1002/spp2.1552)
Supplement: Appendix S1 [file EMS196232-supplement-Appendix_S1.pdf]

Supporting Information

Rostral and body shape analyses reveal cryptic diversity of Late Jurassic batomorphs (Chondrichthyes, Elasmobranchii) from Europe

Julia Türtscher, Patrick L. Jambura, Eduardo Villalobos-Segura, Faviel A. López-Romero, Charlie J. Underwood, Detlev Thies, Bruce Lauer, René Lauer, Jürgen Kriwet

Content

Figure S1 2

Figure S2 3

Figure S3 4

Figure S4 4

Figure S5 5

R code 6

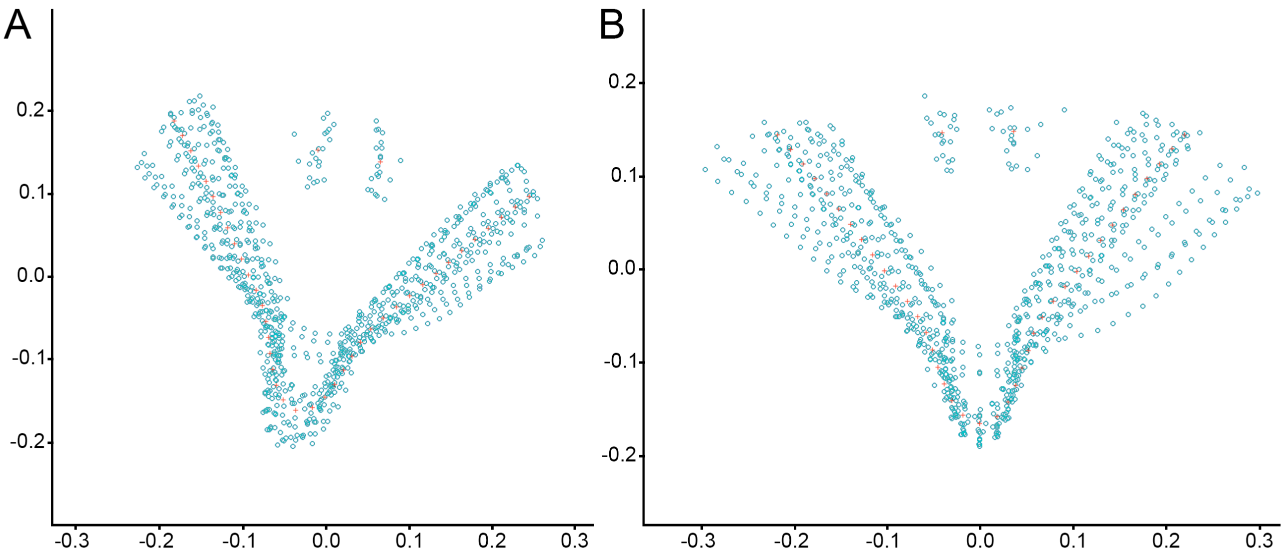

FIG. S1 Geometric Morphometrics: Result of two different superimposition methods; A, Procrustes superimposition; B, RFTRA superimposition. Blue circles indicate landmarks, red crosses indicate landmark centroids. The axes represent the relative position of the landmarks after superimposition.

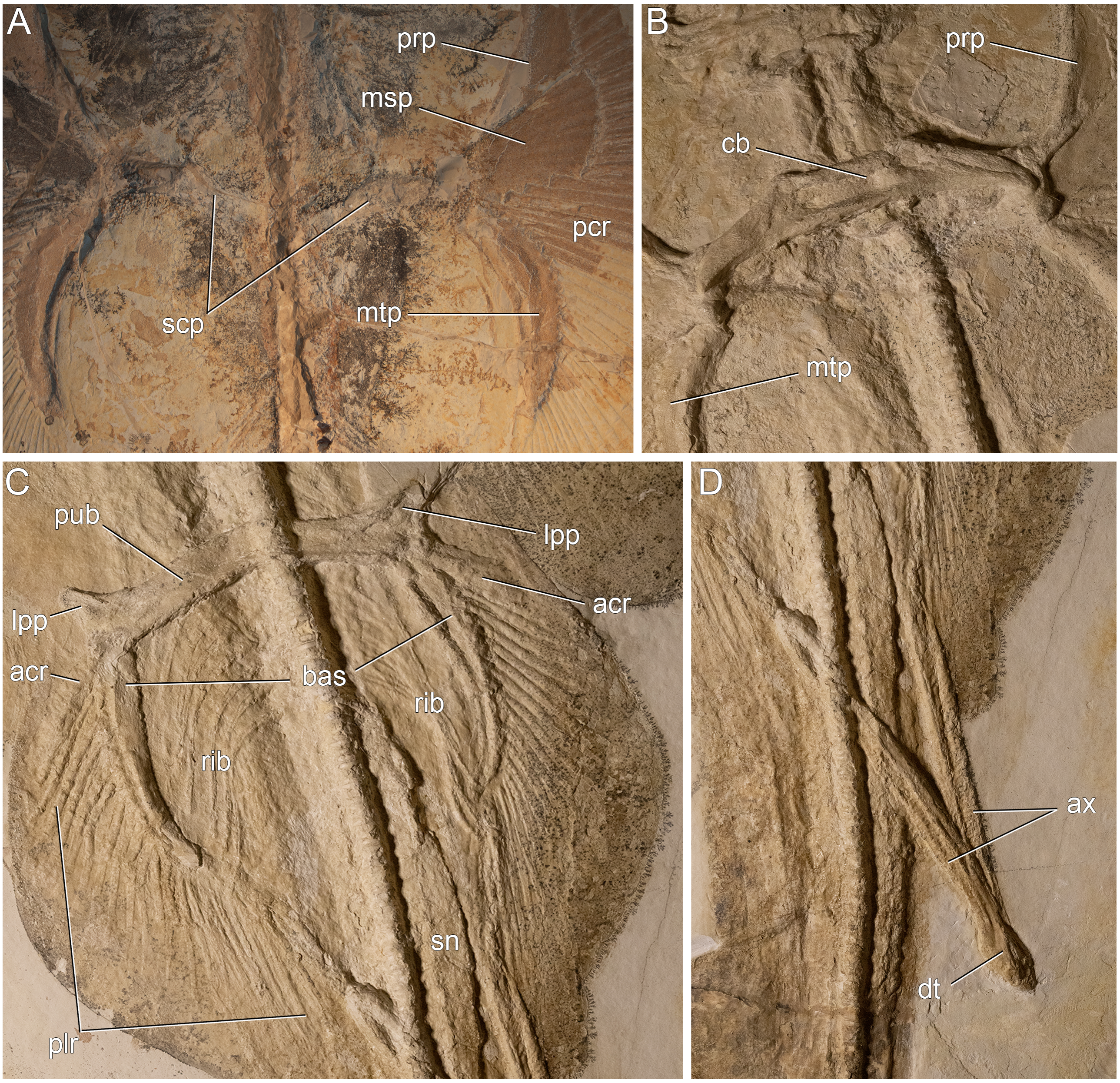

FIG. S2 *Aellopobatis bavarica* gen. et sp. nov.; A, MB 14-12-22-2, pectoral girdle (dorsal view); B, LF 2323, pectoral girdle (ventral view); C, LF 2323, pelvic girdle; C, LF 2323, clasper. *Abbreviations*: **acr**, anterior compound radial; **ax**, axial cartilage; **bas**, basipterygium; **cb**, coracoid bar; **dt**, dorsal terminal cartilage; **lpp**, lateral prepelvic processes; **msp**, mesopterygium; **mtp**, metapterygium; **pcr**, pectoral radials; **plr**, pelvic radials; **prp**, propterygium; **pub**, puboischiadic bar; **rib**, ribs; **scp**, scapular processes; **sn**, supraneural spines. Not to scale.

A

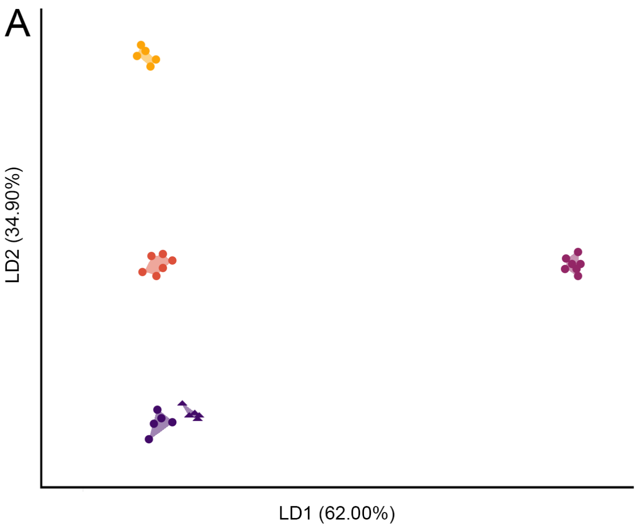

B

	A. bavarica #1	*A. bavarica* #2	*S. bugesiacus*	*As. platypterus*	*B. sismondae*
A. bavarica #1	5	1	0	0	0
A. bavarica #2	3	2	0	0	0
S. bugesiacus	0	0	7	0	0
As. platypterus	0	0	0	6	0
B. sismondae	0	0	0	0	6

FIG. S3 Traditional Morphometrics: Results of the LDA with the two putative *A. bavarica* clusters assigned to two different groups (*A. bavarica* #1 and *A. bavarica* #2). A, Morphospace occupation; B, Leave-one-out cross-validation table.

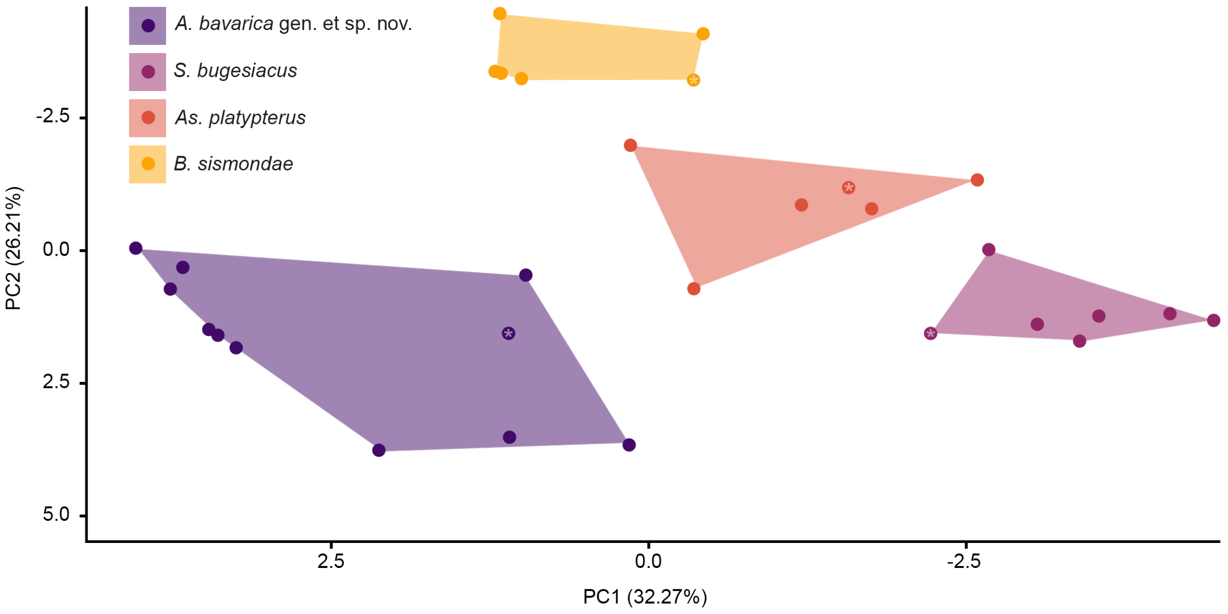

FIG. S4 Traditional Morphometrics: Results of the PCA performed on normally distributed values only, with each measurement adjusted as percentage of the disc width (DW) of each individual; the morphospace is plotted on PC1 (32.27%) and PC2 (26.21%); asterisks indicate the holotype of the respective species.

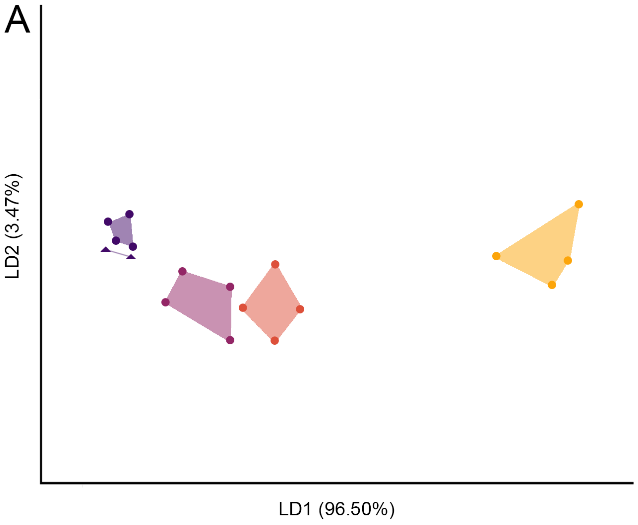

B

	A. bavarica #1	A. bavarica #2	S. bugesiacus	As. platypterus	B. sismondae
A. bavarica #1	4	0	0	0	0
A. bavarica #2	2	0	0	0	0
S. bugesiacus	0	0	3	1	0
As. platypterus	0	0	0	4	0
B. sismondae	0	0	0	0	4

FIG. S5 Geometric Morphometrics: Results of the LDA with the two putative *A. bavarica* clusters (recovered in the Traditional Morphometrics morphospace, see Fig. 9A) assigned to two different groups (*A. bavarica* #1 and *A. bavarica* #2). A, Morphospace occupation; B, Leave-one-out cross-validation table.

R code:

```
#####################################################
#         Source Code: Türtscher et al.             #
#   Cryptic diversity of late jurassic batomorphs   #
#                                                   #
#          #1  Traditional Morphometrics            #
#                                                   #
#####################################################

# set the working directory
setwd("/your_working-directory")

# load the packages
library(geomorph)
library(missMDA)
library(FactoMineR)
library(Momocs)
library(ggpubr)

#####################################################
### START: PCA with missing values

# load the data with missing values
TM.missing <- read.csv("TM.missing.csv", header = TRUE,
                           sep = ",", dec = ",",
                           row.names = 1,
                           na.strings = "NA")

measure.miss <- TM.missing[, 1:26]

# estimate the number of dimensions for the PCA by cross-validation
ndim <- estim_ncpPCA(measure.miss, scale = TRUE)
ndim

ndim$ncp

# impute the missing values of a dataset with the PCA model
comp <- imputePCA(measure.miss, ncp = ndim$ncp, scale = TRUE)
comp

imp <- cbind.data.frame(comp$completeObs)
imp

write.csv(imp,"/your_directory/your_filename.csv", row.names = FALSE)

miss.pca <- PCA(comp$completeObs)
summary(miss.pca)

### END: PCA with Missing Values
#####################################################

# load the data without missing values
TM_JurBat <- read.table("TM-JurBat.csv", header = TRUE,
                     sep = ",", row.names = 1, dec = ",")

measure <- TM_JurBat[, 1:26]
binary  <- TM_JurBat[, 27:31]
mix <- TM_JurBat[, 1:31]
scd <- scale(measure)
smd <- cbind(scd, binary)

# Principal Component Analysis (PCA)
# Table S4
TM.PCA         <- prcomp(measure, scale. = TRUE)
Summary.TM.PCA <- summary(TM.PCA)
```

```
Summary.TM.PCA

TM.taxa   <- as_PCA(TM.PCA, TM_JurBat[, 34])
TM.factor.taxa   <- factor(TM_JurBat$taxa)

plot_PCA(TM.taxa, f = TM.factor.taxa,
         palette = pal_manual(c("#420a68","#dd513a","#fca50a",
                                "#EC7B22","#932667"))
) %>% layer_points(cex = 1.5)

# Figure 9A
Y <- ordinate(measure[,1:26], scale. = TRUE)
summary(Y)

TM.gdf <- data.frame(taxa = TM_JurBat$taxa,
                     ID   = TM_JurBat$ID,
                     PC1  = Y$x[,1],
                     PC2  = Y$x[,2],
                     PC3  = Y$x[,3])

TM.gdf$taxa <- factor(TM.gdf$taxa,
                      levels=c("Aellopobatis",
                               "Spathobatis",
                               "Asterodermus",
                               "Belemnobatis"),
                      ordered = TRUE)

ggscatter(TM.gdf, x = "PC1", y = "PC2",
          color = "taxa", size = 2,
          palette = c("#420a68","#932667",
                      "#dd513a","#fca50a"),
          ellipse = TRUE, ellipse.type = "convex", ellipse.alpha = 0.5,
          mean.point = FALSE, ellipse.border.remove = TRUE,
          xlab = "PC1 (33.37%)", ylab = "PC2 (27.05%)",
          legend.title = "Species")+
  font("legend.text", face = "italic")

# Figure 9B
# Table S5
loadings <- TM.PCA$rotation
loadings # safe loadings in csv-file (rows: PC1 & PC2, columns: measurements)

loadings.all <- read.table("loadings.csv", header = TRUE,
                           sep = ",", row.names = 1, dec = ",")

library(tidyr)
loadings.long <- pivot_longer(data = loadings.all,
                              cols = c(1:26),
                              values_transform = as.numeric,
                              names_to = "Measurement",
                              values_to = "Loading")

write.csv(loadings.long,"/your_directory/loadings_long.csv", row.names = FALSE)

loadings_PC1_PC2 <- read.table("loadings_long.csv", header = TRUE,
                               sep = ",", row.names = 1, dec = ",")

loadings.df <- data.frame(Measurement = loadings_PC1_PC2$Measurement,
                          Loading     = loadings_PC1_PC2$Loading,
                          PC          = loadings_PC1_PC2$PC,
                          Color       = loadings_PC1_PC2$Color)
                          # PC & Color were added manually

loadings.df$Measurement <- factor(loadings.df$Measurement,
```

```
                                  levels=c("TL","SMAX","DL","HL","SPV",
                                           "NC","JW","MAXR","MINR","RL",
                                           "RAD","PCGW","PCPV","PVGW",
                                           "PVCF","PVL","MDMET","MDBAS",
                                           "MDMETO","MDBASO","MWMET","MWBAS",
                                           "LMET","LBAS","MAXWMES","HDW"),
                                  ordered = TRUE)

barplot <- ggplot(data = loadings_PC1_PC2) +
  geom_bar(aes(x = loadings.df$Measurement, y = Loading,
               fill = Color),
           position = "stack", stat = "identity")+
  theme(legend.position = "none", axis.text.x = element_text(angle = 90))+
  facet_wrap(~PC, strip.position = "bottom")+
  xlab("Measurement") + ylab("Loadings")

barplot

# create a dataframe
TM.data <- data.frame(taxa = TM_JurBat$taxa, ID = TM_JurBat$ID,
                      group = TM_JurBat$grouping,
                      TL = TM_JurBat$TL, SMAX = TM_JurBat$SMAX,
                      DL = TM_JurBat$DL, HL = TM_JurBat$HL,
                      SPV = TM_JurBat$SPV, NC = TM_JurBat$NC,
                      JW = TM_JurBat$JW, MAXR = TM_JurBat$MAXR,
                      MINR = TM_JurBat$MINR, RL = TM_JurBat$RL,
                      RAD = TM_JurBat$RAD, PCGW = TM_JurBat$PCGW,
                      PCPV = TM_JurBat$PCPV, PVGW = TM_JurBat$PVGW,
                      PVCF = TM_JurBat$PVCF, PVL = TM_JurBat$PVL,
                      MDMET = TM_JurBat$MDMET, MDBAS = TM_JurBat$MDBAS,
                      MDMETO = TM_JurBat$MDMETO, MDBASO = TM_JurBat$MDBASO,
                      MWMET = TM_JurBat$MWMET, MWBAS = TM_JurBat$MWBAS,
                      LMET = TM_JurBat$LMET, LBAS = TM_JurBat$LBAS,
                      MAXWMES = TM_JurBat$MAXWMES, HDW = TM_JurBat$HDW)

TM.data$taxa <- factor(TM.data$taxa,
                                  levels=c("Aellopobatis",
                                           "Spathobatis",
                                           "Asterodermus",
                                           "Belemnobatis"),
                                  ordered = TRUE)

# Figure 10
ggplot(data = TM.data, aes(x = taxa, y = JW, fill = taxa))+
  geom_boxplot(outlier.shape = NA, alpha = 0.5,
               fill = c("#420a68","#932667","#dd513a","#fca50a"))+
  geom_point(shape = 21, size = 1.5,
             position = position_dodge(0.92),
             color = "black", alpha = 0.5,
             aes(fill = factor(taxa)))+
  scale_fill_manual(values = c("#420a68","#932667","#dd513a","#fca50a"))+
  theme_minimal()+
  theme_linedraw(base_size = 14)+
  theme(legend.position = "none",
        axis.text.x = element_text(face = "italic"))+
  xlab("Species") + ylab("JW")

# Linear Discriminant Analysis (LDA)
JurBat.SMD <- prcomp(smd, scale. = TRUE)

SMD.taxa    <- as_PCA(JurBat.SMD, TM_JurBat[, 34])
SMD.group   <- as_PCA(JurBat.SMD, TM_JurBat[, 35])
Factor.taxa <- factor(TM_JurBat$taxa)
Factor.group  <- factor(TM_JurBat$grouping)
```

```
JurBat.LDA <- LDA(SMD.taxa, Factor.taxa, retain = 0.99)

# Table S2
Tax.LDA <- LDA(SMD.taxa, Factor.taxa, retain = 0.99)
Tax.LDA

# Figure S3
Group.LDA <- LDA(SMD.group, Factor.group, retain = 0.99)

plot_LDA(Group.LDA,
         palette = pal_manual(c("#420a68","#420a68",
                                "#dd513a", "#fca50a","#932667")),
         zoom = 1.3) %>% layer_points(cex = 1.5)

# Table S6
Group.LDA

# Shapiro-Wilk test for normal distribution (H0 = normal distribution)
# Table S7
shapiro.test(TM_JurBat$HL)

### non-normally distributed:
# Kruskal-Wallis test (H0 = equality)
# Table S8
kruskal.test(TM_JurBat$HL~TM_JurBat$taxa)

# Post hoc pairiwse Wilcoxon test (H0 = equal medians)
# Table S9
pairwise.wilcox.test(TM_JurBat$HL, TM_JurBat$taxa, paired = FALSE,
                     p.adjust = "bonferroni")

### normally distributed:
# ANOVA
# Table S10
JurBat.anova <- lm.rrpp(TL~taxa,
                        data = TM.data,
                        print.progress = FALSE, iter = 999)

anova(JurBat.anova)

# pairwise comparisons
# Table S11
Taxa.PW <- pairwise(JurBat.anova , groups = TM.data$taxa,
                    print.progress = FALSE)

summary(Taxa.PW, test.type = "dist", confidence = 0.95,
        stat.table = TRUE)

### subsample with only normally distributed values
normal.sub <- TM_JurBat[, c(1:3,5,7:8,10:12,14:19,21:24,26)] # measurements only
normal.sub <- droplevels(normal.sub)
normal.sub

normal.class.sub <- TM_JurBat[, c(1:3,5,7:8,10:12,14:19,21:24,26)] # with
classifiers

# PCA with normally distributed values
# Table S12
normal.pca <- prcomp(normal.sub, scale. = TRUE)
summary(normal.pca)

# Table S13
normal.loadings <- normal.pca$rotation
```

```
normal.loadings

# Figure S4: PCA with normally distributed values
Y.norm <- ordinate(normal.sub[,1:20], scale. = TRUE)
summary(Y.norm)

normal.gdf <- data.frame(taxa     = TM_JurBat$taxa,
                         ID       = TM_JurBat$ID,
                         PC1      = Y.norm$x[,1],
                         PC2      = Y.norm$x[,2],
                         PC3      = Y.norm$x[,3])

normal.gdf$taxa <- factor(normal.class.sub$taxa,
                             levels=c("Aellopobatis",
                                      "Spathobatis",
                                      "Asterodermus",
                                      "Belemnobatis"),
                          ordered = TRUE)

ggscatter(normal.gdf, x = "PC1", y = "PC2",
          color = "taxa", size = 2,
          palette = c("#420a68","#932667",
                      "#dd513a","#fca50a"),
          xlim = c(4,-4.5),
          ylim = c(5,-4.5),
          ellipse = TRUE, ellipse.type = "convex", ellipse.alpha = 0.5,
          mean.point = FALSE, ellipse.border.remove = TRUE,
          xlab = "PC1 (32.27%)", ylab = "PC2 (26.21%)",
          legend.title = "Species")+
  scale_x_reverse()+
  scale_y_reverse()+
  font("legend.text", face = "italic")



#####################################################
#         Source Code: Türtscher et al.             #
#   Cryptic diversity of late jurassic batomorphs   #
#                                                   #
#           #2  Geometric Morphometrics             #
#                                                   #
#####################################################

# set the working directory
setwd("/your_working-directory")

# load the packages
library(geomorph)
library(Momocs)
library(ggpubr)

# load the data
GM_JurBat <- readland.tps("GM-JurBat.tps",
                       specID = "imageID",
                       readcurves = TRUE, warnmsg = TRUE)

GM_JurBat_Class <- read.csv("GM-JurBat-Class.csv",
                         header = TRUE, sep = ",")

GM_JurBat_Sliders <- as.matrix(read.csv("GM-JurBat-Sliders.csv",
                                    header = TRUE))

### Generalized Procrustes Analysis (GPA)
GM_JurBat_GPA <- gpagen(GM_JurBat, curves = GM_JurBat_Sliders,
```

```
                    ProcD = FALSE, # if FALSE = bending energy
                    print.progress = FALSE)

plotAllSpecimens(GM_JurBat_GPA$coords)

### Principal Component Analysis (PCA)
# Table S14
GM_JurBat_PCA <- gm.prcomp(GM_JurBat_GPA$coords, scale = FALSE)
summary(GM_JurBat_PCA)

# create a geomorph data frame
GM_JurBat_gdf <- geomorph.data.frame(shape      = GM_JurBat_GPA$coords,
                                     size       = log(GM_JurBat_GPA$Csize),
                                     ID         = GM_JurBat_Class$ID,
                                     country    = GM_JurBat_Class$country,
                                     taxa       = GM_JurBat_Class$taxa,
                                     group      = GM_JurBat_Class$group,
                                     ExKim      = GM_JurBat_Class$include)

# Figure 11
GM_JurBat_df <- data.frame(tax = GM_JurBat_Class$taxa,
                           ID  = GM_JurBat_Class$ID,
                           PC1 = GM_JurBat_PCA$x[,1],
                           PC2 = GM_JurBat_PCA$x[,2],
                           PC3 = GM_JurBat_PCA$x[,3])

GM_JurBat_df$tax <- factor(GM_JurBat_df$tax,
                           levels = c("Aellopobatis",
                                      "Spathobatis",
                                      "Kimmerobatis",
                                      "Asterodermus",
                                      "Belemnobatis"),
                           ordered = TRUE)

ggscatter(GM_JurBat_df, x = "PC1", y = "PC2",
          color = "tax",
          palette = c("#420a68","#932667","#EC7B22","#dd513a","#fca50a"),
          ellipse = TRUE, ellipse.type = "convex",
          ellipse.border.remove = TRUE,
          ellipse.alpha = 0.4,
          xlab = "PC1 (80.39%)", ylab = "PC2 (14.39%)",
          legend.title = "Species") +
  font("legend.text", face = "italic")

# Size - Shape
GM_size_df <- data.frame(PC1   = GM_JurBat_PCA$x[,1],
                         PC2   = GM_JurBat_PCA$x[,2],
                         ID    = GM_JurBat_Class$ID,
                         taxa  = GM_JurBat_Class$taxa)

GM_size <- procD.lm(shape~size, data = GM_JurBat_gdf,
                              print.progress = FALSE)
# Table S16
summary(GM_size)

# Figure 12
col5        <- c("#3B0964FF","#E55C30FF","#FCA309FF","#E48521","#9F2A63FF")
names(col5) <- levels(GM_JurBat_gdf$taxa)
col5.gp     <- col5[match(GM_JurBat_Class$taxa, names(col5))]

plot(GM_size, type = "regression", reg.type = "RegScore",
     predictor = GM_JurBat_gdf$size, pch = 21, col = col5.gp,
     bg = col5.gp)
```

```r
### Procrustes ANOVA
GM_Taxa_aov <- procD.lm(shape~taxa, data = GM_JurBat_gdf,
                     RRPP = TRUE, print.progress = FALSE)

# Table S17
summary(GM_Taxa_aov)

# pairwise comparisons
GM_Taxa_PW <- pairwise(GM_Taxa_aov, groups = GM_JurBat_gdf$taxa,
                    print.progress = FALSE)

# Table S18
summary(GM_Taxa_PW, test.type = "dist", confidence = 0.95,
        stat.table = TRUE)

### subset without Kimmerobatis etchesi
GM_ExKim_GPA   <- GM_JurBat_GPA$coords[,,c(1:20)]
GM_ExKim_Class <- GM_JurBat_Class[c(1:20),]

GM_ExKim_sub <- subset(GM_ExKim_Class, include == "INC",
                       select = c("taxa","group","ID"))

GM_ExKim_sub <- droplevels(GM_ExKim_sub)
GM_ExKim_PCA <- gm.prcomp(GM_ExKim_GPA)
summary(GM_ExKim_PCA)

GM_ExKim_df  <- data.frame(Tax  = GM_ExKim_sub$taxa,
                           ID   = GM_ExKim_sub$ID,
                           grp  = GM_ExKim_sub$group,
                           PC1  = GM_ExKim_PCA$x[,1],
                           PC2  = GM_ExKim_PCA$x[,2],
                           PC3  = GM_ExKim_PCA$x[,3])

GM_ExKim_df$Tax <- factor(GM_ExKim_df$Tax,
                          levels = c("Aellopobatis",
                                     "Spathobatis",
                                     "Asterodermus",
                                     "Belemnobatis"),
                          ordered = TRUE)

ggscatter(GM_ExKim_df, x = "PC1", y = "PC2",
          color = "Tax", size = 2,
          palette = c("#420a68","#932667","#dd513a","#fca50a"),
          ellipse = TRUE, ellipse.type = "convex",
          ellipse.border.remove = TRUE,
          ellipse.alpha = 0.5,
          xlab = "PC1", ylab = "PC2",
          legend.title = "Taxa") +
  font("legend.text", face = "italic")

# Linear discriminant analysis (LDA) without Kimmerobatis etchesi
GM_ExKim.pca    <- as_PCA(GM_ExKim_PCA, GM_ExKim_Class[, 4])
GM_ExKim_group  <- factor(GM_ExKim_Class$group)
GM_ExKim_taxa   <- factor(GM_ExKim_Class$taxa)

GM_ExKim_lda <- LDA(GM_ExKim.pca, GM_ExKim_taxa, retain = 0.95)

plot_LDA(GM_ExKim_lda,
         palette = pal_manual(c("#420a68","#dd513a","#fca50a","#932667")),
         zoom = 1.3) %>% layer_points(cex = 1.5)

# Table S3
GM_ExKim_lda # obtain LOOCV table
```

```
# LDA on putative morphotypes of A. bavarica:
LDA.gpa   <- GM_JurBat_GPA$coords[,,c(1:2,4:7,9:20)]
LDA.Class <- GM_JurBat_Class[c(1:2,4:7,9:20),]

LDA.sub <- subset(LDA.Class, include_group == "INC",
                  select = c("taxa","group","ID"))

LDA.sub <- droplevels(LDA.sub)
LDA.PCA <- gm.prcomp(LDA.gpa)
LDA.df  <- data.frame(Tax = LDA.sub$taxa,
                      ID  = LDA.sub$ID,
                      grp = LDA.sub$group,
                      PC1 = LDA.PCA$x[,1],
                      PC2 = LDA.PCA$x[,2],
                      PC3 = LDA.PCA$x[,3])

LDA.df$grp <- factor(LDA.df$grp,
                     levels=c("Aellopobatis_1",
                              "Aellopobatis_2",
                              "Spathobatis",
                              "Asterodermus",
                              "Belemnobatis"),
                     ordered = TRUE)

LDA.pcaM <- as_PCA(LDA.PCA, LDA.Class[, 4])
grp.factor <- factor(LDA.Class$group)

plot_PCA(LDA.pcaM, f = grp.factor,
         palette =
pal_manual(c("#420a68","#420a68","#dd513a","#fca50a","#932667"))
) %>% layer_points(cex = 1.5)

LDA.lda <- LDA(LDA.pcaM, grp.factor, retain = 0.95)

# Figure S5
plot_LDA(LDA.lda,
         palette =
pal_manual(c("#420a68","#420a68","#dd513a","#fca50a","#932667")),
         zoom = 1.3) %>% layer_points(cex = 1.5)

# Table S15
LDA.lda

#####################################################
#                                                   #
#                         END                       #
#                                                   #
#####################################################
```
